# Efficacy and Safety of Cannabis Extracts for the Treatment of Osteoarthritis: A Systematic Review and Meta‐Analysis of Preclinical and Human Studies

**DOI:** 10.1155/prm/3998239

**Published:** 2026-07-27

**Authors:** Bedru J. Abafita, Ambrish Singh, Dawn Aitken, Benny Antony

**Affiliations:** ^1^ Menzies Institute for Medical Research, University of Tasmania, Hobart, Australia, utas.edu.au

**Keywords:** cannabis extract, human, osteoarthritis, preclinical, systematic review

## Abstract

**Background:**

Interest in cannabis extracts for chronic pain, including osteoarthritis (OA), is growing. This systematic review evaluated the efficacy of cannabis extracts in OA across preclinical and clinical studies.

**Methods:**

A comprehensive literature search was performed using Medline via Ovid, Embase via Ovid, CINAHL, and Cochrane Central Register of Controlled Trials database to identify studies published up to 2024. Studies assessing the efficacy and safety of cannabis extracts for OA in preclinical and clinical settings were included. Two researchers independently evaluated the risk of bias using the Cochrane Risk of Bias 2 tool and Office of Health Assessment and Translation (OHAT) risk of bias tool.

**Results:**

Twenty‐six studies (20 preclinical and 6 clinical) met the inclusion criteria. Most preclinical studies (*n* = 20) examined the effects of cannabis extracts in preclinical animal models of OA and domestic dogs with spontaneous OA, assessing structural changes (*n* = 8), proinflammatory modulation (*n* = 9), and pain (*n* = 13). Preliminary findings indicated that cannabis extracts exerted chondroprotective, chondrogenerative, anti‐inflammatory, and analgesic effects in OA models, reducing hyperalgesia and mechanical/thermal allodynia. Six clinical studies investigated pain, quality of life (QoL), and function. Four randomized controlled trials (RCTs) found no significant benefits in pain relief, QoL, or functional improvement with cannabis extracts. Adverse effects were generally mild.

**Conclusion:**

Preclinical evidence provides preliminary, low‐certainty support for the anti‐inflammatory, analgesic, and chondroprotective properties of cannabinoid‐based therapies in OA. Current clinical evidence remains insufficient to establish efficacy for pain or function. Further high‐quality, large‐scale RCTs are necessary to confirm the efficacy and clarify the safety profile of cannabis extracts in OA management.

## 1. Introduction

Osteoarthritis (OA) is a chronic musculoskeletal disorder that affects more than 595 million people worldwide and about 51.8 million adults in the United States [1–3]. OA is the most common type of arthritis leading to knee and hip replacement surgery [2, 4]. OA was ranked 17th most prevalent chronic disease worldwide and accounted for over 8.9 million years lived with disability (YLDs) in 1990, rising to over 21.3 million YLDs in 2021 [5]. Given the substantial costs associated with joint replacement surgery, and the use of prescription medications to manage moderate to severe pain, OA exerts a significant economic burden on healthcare systems and individuals [6].

The current treatment options for OA are unsatisfactory. Pharmacological treatments, such as nonsteroidal anti‐inflammatory drugs (NSAIDs) and corticosteroids, are only modestly effective for pain [7]. Surprisingly, paracetamol (one of the most commonly used analgesics for OA) was found to have a minor, nonclinically significant effect on pain [8, 9]. Pain control in OA patients remains poor, with more than 75% requiring additional symptomatic treatment, leading to surgical treatment with costly joint replacements [10]. In addition, commonly prescribed drugs can cause gastrointestinal, renal and cardiovascular complications, making them contraindicated in many OA patients due to their high rate of comorbidities [11]. While opioids are not recommended for the treatment of OA [12–14], they are commonly used to treat chronic pain associated with OA [15, 16]. Data from Australia have shown a significant increase in opioid prescribing for people with hip OA, increasing from 19.7% in the period 2005–2010 to 25.6% in the period 2010–2016. Similarly, for knee OA, opioid prescribing increased from 10.3% to 14.9% during the same time periods [17]. This increase in opioid prescription was accompanied by marked increases in rates of opioid use disorder and drug overdose mortality [18, 19]. Thus, there is an urgent need for safer and more effective therapies.

For over 4000 years, the Cannabis sativa plant has been documented in traditional Chinese medicine as a therapeutic agent to alleviate joint pain and systemic inflammation. Similarly, historical Ayurvedic medicine incorporates various complex formulations, including specific cannabis extracts, for the holistic management of OA symptoms [20, 21]. While these ancient practices relied primarily on empirical observation, their long‐standing use highlights a historical precedent for targeting chronic musculoskeletal pain pathways via plant‐derived compounds. This extensive ethnobotanical history serves as a critical foundation for modern pharmacological interest, bridging ancient therapeutic wisdom with contemporary evidence‐based research into the plant’s medicinal efficacy.

Recently, there is increasing interest in the use of various cannabis extracts for the treatment of chronic pain conditions, including OA. Therapeutic Goods Administration (TGA) of Australia has recently given conditional approval to move cannabidiol (CBD) as a Schedule 3 drug for up to a maximum recommended daily dose of 150 mg [22]. TGA has already assessed low‐dose CBD as a safe ingredient, hence downscheduling it from prescription‐only to an over‐the‐counter product [23]. However, the product needs to be listed on the Australian Register for Therapeutic Goods (ARTG) to be sold as an over‐the‐counter CBD product [22]. Review of the Australian New Zealand Clinical Trials Registry (ANZCTR), an online registry of clinical trials, shows very few trials are currently underway investigating cannabinoids that could relate to a Schedule 3 registration program.

There is increasing evidence on the mechanism of action of cannabis for the treatment of OA. Studies have identified two cannabinoid receptors, cannabinoid 1 receptor (CB1R) and cannabinoid 2 receptor (CB2R) [24, 25]. Cannabis species has a variety of cannabinoids, but the two that are present in the highest concentrations are delta‐9‐tetrahydrocannabinol (THC) and CBD, which have anti‐inflammatory and analgesic properties that work by interacting with cannabinoid receptors in the body, which are part of the endocannabinoid system (ECS) [26]. Cannabinoids’ antinociceptive pathways are distinct from those of other medications already in use, which opens a new promising option for pain management, particularly for inflammatory pain that does not respond to available pharmacological treatment [27].

Cannabinoid receptors were found to play a role in peripheral pain signaling in preclinical studies; CB2R agonists demonstrated suppression of capsaicin‐induced Ca^2+^ influx via reduction of cyclic adenosine monophosphate (cAMP), which resulted in transient receptor potential vanilloid 1 (TRPV1) desensitization [28]. The potential for cannabinoids to modulate chronic pain via multiple synergistic targets is demonstrated by the increased mRNA expression of the orphan G‐protein–coupled receptor activity GPR18, GPR55, and CB2R in the spinal cord and dorsal root ganglion (DRG) [29, 30].

In recent years, several preclinical and human studies suggest that cannabis extracts may help to reduce pain and inflammation and that they may have disease‐modifying effects in OA [31–34]. Due to their acceptable safety profile and growing clinical interest, cannabinoids have been explored as a candidate therapy for OA warranting further investigation. Therefore, this systematic review aims to synthesize the available evidence on the safety and efficacy of cannabinoids for the treatment of OA, including both preclinical and clinical studies.

## 2. Methods

### 2.1. Search Strategy

This systematic review was conducted and reported in accordance with the Preferred Reporting Items for Systematic Reviews and Meta‐Analyses (PRISMA) recommendation [35]. This study is registered on PROSPERO (registration number: CRD42024539941). We searched the following databases from inception to April 2024. The complete search strategies for each database are provided in Supporting Appendix 1:

1. EMBASE via Ovid
2. CINAHL
3. MEDLINEvia Ovid
4. The Cochrane Central Register of Controlled Trials

### 2.2. Additional Searches

To supplement the literature search, additional searches were conducted in the Scopus and Web of Science databases. A manual search of reference lists from all retrieved studies and abstracts from conference proceedings of major international organizations involved in OA research such as the European League Against Rheumatism (EULAR), Osteoarthritis Research Society International (OARSI), and the American College of Rheumatology (ACR) was also performed for the past 2 years. Finally, a clinical trial registry database and gray literature search was conducted to reduce the risk of publication bias.

### 2.3. Study Eligibility

Two reviewers (Bedru J. Abafita and Ambrish Singh) independently screened the eligibility of studies based on their title and abstract, followed by full‐text screening using Covidence software. Any inconsistency and disagreement between the two reviewers were resolved by the third reviewer. Eligible studies included human clinical studies and preclinical animal or cell‐based studies assessing cannabinoid‐based therapies for OA. For human research, randomized controlled trials (RCTs) assessing the safety and efficacy of cannabis extracts in OA were included, including RCTs with parallel‐group, crossover, and open‐label designs. For preclinical studies, any cannabis extracts administered to investigate their effect on OA in a domestic animal OA model were included. We included full journal publication and conference abstracts with sufficient data for analysis. The efficacy outcome of interest included pain, physical function, and quality of life (QoL). Safety outcomes included adverse events (AEs) and serious adverse events (SAEs) associated with cannabis extracts. The outcomes of interest in preclinical studies included pain‐related behavioral assessments, OA‐related inflammatory markers, and structural changes in joint tissues. Exclusion criteria included non‐English articles, studies using undefined cannabis extracts, nonclinical or nonexperimental designs, and those lacking relevant outcome measures.

### 2.4. Types of Interventions

The interventions considered for inclusion were cannabinoid‐based therapies, including plant‐derived phytocannabinoids (such as CBD, THC, and cannabigerol [CBG]) as well as synthetic cannabinoid receptor agonists (such as WIN‐55,212‐2, HU308, and JWH‐133). Endogenous cannabinoids (endocannabinoids produced naturally by the body) and metabolic enzyme inhibitors were excluded from this review.

### 2.5. Data Extraction

Two reviewers (Bedru J. Abafita and Ambrish Singh) independently extracted data from each included preclinical and human study and any inconsistencies were reviewed by a third reviewer (Benny Antony). For human studies, relevant information, such as authors, year of publication, study design, population characteristics, intervention and comparator details, duration of follow‐up, type of OA, efficacy, and safety outcome measures were extracted using a standard extraction template. A similar extraction form was used for preclinical studies and included author, year of publication, animal characteristics, OA model, cell phenotype, intervention dose, frequency, and outcome measures. For studies with incomplete or unavailable data, corresponding authors were contacted directly. Where no response was received, data were sourced from previous systematic reviews that included the missing primary trial data. Graphically presented outcomes were extracted using WebPlotDigitizer [36].

Standard deviations (SDs) were extracted when reported. If not provided, SDs were calculated from standard errors or confidence intervals (CIs). For change scores (SDdiff), SDs were calculated using the baseline SD (SDbl) and postintervention SD (SDpi), applying a conservative correlation coefficient (*r* = 0.5), as recommended by the Cochrane Handbook [37]. For studies with more than two arms, we combined two intervention groups into a single intervention group and analyzed it with the independent comparator arms to enable comparison.

### 2.6. Risk of Bias and Certainty Assessment

The included studies were evaluated for risk of bias and certainty of evidence independently by two reviewers (Bedru J. Abafita and Ambrish Singh), and if necessary, any disagreement in each domain was resolved by the third reviewer (Benny Antony). The risk of bias and certainty of evidence in human studies was assessed using the Cochrane risk of bias tool (RoB2) and GRADEpro GDT, respectively [38, 39]. Certainty of evidence was graded “high,” “moderate,” “low,” or “very low.” The reason for upgrading or downgrading the certainty of evidence was explained in a footnote. For preclinical studies, the Office of Health Assessment and Translation (OHAT) risk of bias rating tool was used [40].

### 2.7. Data Analysis

A random‐effects model was employed to account for heterogeneity among studies. Treatment effect sizes for pain were calculated using the standardized mean difference (SMD) with 95% CIs, as pain was measured on a continuous scale in both human and preclinical studies. Effect sizes were categorized according to Cohen’s criteria, where an SMD of 0.2 indicates a small effect, 0.5 a medium effect, and 0.8 a large effect [41]. We utilized change scores from baseline rather than end‐of‐study scores to account for interpatient variability. If authors reported only postintervention values, we combined these with change scores according to the approach suggested by the Cochrane Handbook [37]. For dichotomous outcomes, such as AEs, Mantel–Haenszel statistics were employed to calculate risk differences with 95% CIs. Heterogeneity was assessed using the *I* ^2^ statistic. We used a narrative synthesis approach to present the results of outcomes where data were not available or suitable for meta‐analysis (physical function, QoL and structural outcomes, and proinflammatory factors). All statistical analyses were performed using STATA Version 18, with results presented as forest plots.

## 3. Results

**FIGURE 1:**
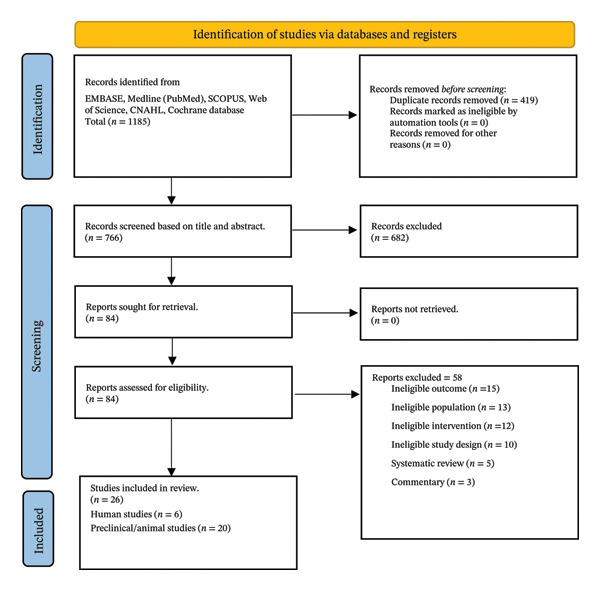
Preferred reporting items for systematic reviews and meta‐analyses (PRISMA) flow diagram of the search algorithm.

A total of 1185 studies were identified from database searches. After the removal of duplicates, 764 studies remained for the title and abstract screening (Figure 1).

Following abstract and title screening, 83 studies remained for full‐text review. Finally, 26 studies met the inclusion criteria and were included (20 preclinical and 6 clinical studies) in this systematic review.

### 3.1. Preclinical Studies

#### 3.1.1. Overall Description of Included Studies

Twenty preclinical studies involving 845 rodents (61% mice, 39% rats) were included. The rodent sex distribution was 47% male, 43% female, and 10% not reported. Additionally, 107 domestic dogs with spontaneous OA were included, with a sex ratio of 49.5% female and 50.5% male, with a mean age of 11 years. These studies investigated the effects of various cannabis extracts on OA [31–34, 42–57]. Five studies evaluated the effect of cannabis extracts: One study [49] used rat chondrocytes, while four studies [45, 46, 50, 54] used human chondrocytes. Additionally, five studies used both animal OA models and chondrocyte cells [31–33, 44, 51], five studies used domestic animal with spontaneous OA [34, 42, 47, 48, 52], and five studies focused solely on animal OA models [43, 53, 55–57]. Among these, nine studies examined the effects of CBD [31, 34, 42, 48, 49, 52–54, 57], two studies investigated the effect of THC [32, 33], and six studies investigated the effects of CB2 receptor agonists [43, 44, 51, 54–56], while the remaining three studies investigated the effects of CB1 receptor agonists [45, 46, 50].

Preclinical studies were conducted on healthy mice or rats [31–34, 43, 44, 51, 53, 55, 57], except for five studies that used OA models in domestic dogs [34, 42, 47, 48, 52]. Most OA animal models were induced by destabilization of medial meniscus (DMM) surgery [31–33, 44] and induced chemically [32–34, 43, 51, 53, 55, 56], with only one study using noninvasive anterior cruciate ligament rupture (ACLR) [54].

#### 3.1.2. Effect of Cannabis Extracts on Structural Outcomes

Eight preclinical studies examined the effect of different cannabis extracts on structural outcomes in OA [31–33, 44, 46, 49–51], as shown in Table 1 and Supporting Appendix 2. A study using cultured chondrocyte cells treated with poly lactic‐co‐glycolic acid nanoparticles (CBD‐PLGA‐NPs) [49] and CB1 receptor agonist (WIN‐55) [46] showed a significantly attenuated expression of extracellular matrix (ECM) catabolic proteins, including matrix metalloproteinase (MMP‐3 and MMP‐13). Treatment with CB1 receptor agonist (WIN‐55) [50] inhibited A disintegrin and metalloproteinase with thrombospondin motif 4 (ADAMTS‐4) activity in a concentration‐dependent manner both in the presence and absence of interleukin‐1 beta (IL‐1β), whereas CB2 receptor agonists (HU308 and JWH‐133) [44, 51] significantly increased the expression of anabolic protein aggrecan and Type II collagen (COL II) in chondrocyte cells and improved cell viability.

**TABLE 1:** Effects of cannabis extracts on structural changes, inflammatory factors, and pain in preclinical studies.

Author (year)	Cell/tissue source & species	Cell phenotype/OA model	Cannabinoid intervention & regimen	Finding
Structural outcomes				
Cell studies				
Rockel et al. [33] 2022	Human (OA patients)	OA FLS and chondrocyte	THC (0.1–10 mM) for 48 hours.	Increased apoptosis markers at ≥ 2.5 mM THC
Maglaviceanu et al. [32] 2022	Human (OA patients)	Chondrocytes	THC (0–50 mM) for 48 hours.	Significant decrease in cell viability above 10 mM; apoptosis at 2.5 mM
Dunn et al. [46] 2014	Human (OA patients)	Chondrocytes	WIN‐55 (10 ng/mL IL‐1β + 1–10 mM) for 48 h	Time‐ and concentration‐dependent decrease in MMP‐3, MMP‐13, TIMP‐1, and TIMP‐2 gene expression, with significant effects at concentrations ≥ 2.5 mM
Jin et al. [49] 2023	Rats	Chondrocytes	CBD‐PLGA‐NPs (1.5 mg/mL) for 12 hours	Reduced MMP‐13 expression in OA chondrocytes
Carmon et al. [44] 2021	Human (OA patients)	Chondrocytes	HU308 (10 or 100 nM) for 48 hours	Increased anabolic markers (ACAN, COL2) at 100 nM
Malek et al. [51] 2022	Human	Chondrocytes	JWH‐133 (10 μM)	JWH‐133 improved viability and motility of MIA‐treated chondrocytes
Kong et al. [50] 2016	Human	Chondrocytes	WIN‐55 (0.5–8 μM) + IL‐1β + CB1 & CB2 antagonists	WIN‐55 treatment inhibited ADAMTS‐4 activity in a concentration‐dependent manner, achieving about 85% inhibition at 2 μM in unstimulated cells and 70% at 4 μM in IL‐1β‐stimulated cells. It also reduced syndecan‐1 levels by 75% in both unstimulated and IL‐1β‐stimulated cells.
Animal studies				
Rockel et al. [33] 2022	Mice	Surgically induced (DMM) and chemically induced (MIA)	DMM: THC (1, 5 or 10 mg/kg) via intra‐articular injection (1×/week) or oral (5×/week for 9 weeks) MIA: THC (5 or 10 mg/kg) via oral (5×/week for 3 weeks)	Intra‐articular THC accelerated cartilage degeneration and increased synovitis. Oral D9‐THC reduced cartilage degeneration.
Karuppagouder et al. [31] 2022	Mice	Surgical induced (DMM)	Topical CBD oil (20 mg/kg/day) or CBG oil (10 mg/kg/day)	CBG oil reduced cartilage degeneration, preserved chondrocytes, and reduced total volume of the subchondral bone.
Carmon et al. [44] 2021	Mice	Surgical induced (DMM)	HU308 (0.5 μg) intra‐articular (2×/week for 4 weeks)	Intra‐articularly administered HU308 attenuated cartilage damage and osteophyte appearance compared to mice treated with vehicle or left untreated.
Malek et al. [51] 2022	Rats	Chemically induced (MIA)	JWH‐133 was administered via intra‐articular route at the dose of 100 ng per injection every second day for 2 weeks	JWH‐133 treatment restored the protein levels of TIMP1 and COMP in OA‐affected cartilage and reduced the expression of MMPs such as MMP3, MMP9, and MMP13 in the cartilage.
Modulation of proinflammatory factors				
Cell studies				
Verrico et al. [34] 2020	Mouse Human	Mouse macrophage cells, human monocytic THP‐1 cells, human PBMCs, and mouse splenocytes	100 ng/mL CBD in conjunction with LPS and SEB treatment	CBD significantly reduced TNF‐α levels (97% reduction in THP‐1 cells); significant reduction was observed in other cell types.
Jin et al. [49] 2023	Rats	Chondrocytes	1.5 mg/mL CBD‐PLGA‐NPs or LPS (5 nM) for 12 hours	Downregulated IL‐1β, IL‐6, and TNF‐α in LPS‐induced inflammation, significantly reducing their expression levels
Rzeczycki et al. [54] 2021	Human (OA patients)	Human OA FLS, mBMDM	1 μM HU308 + CBD (10 μM) + 10 ng/mL IL‐1β or 10 ng/mL TNF‐α for 48 hours	Treatment with HU308 and CBD inhibited IL‐1β‐induced M1 macrophage polarization in mBMDMs, reducing proinflammatory gene expression (Il1b, Mmp1b, Il6) and increasing the anti‐inflammatory gene Cd206. CB2 agonism significantly suppressed IL‐1β and TNF‐α‐induced upregulation of proinflammatory and catabolic genes (CCL2, MMP1, MMP3, IL6).
Dunn [45] 2012	Human (OA patients)	Chondrocytes	1–10 mM WIN‐55 + 10 ng/mL IL‐1β for 48 hours.	Chondrocytes treated with WIN‐55 and IL‐1β showed significantly reduced IL‐8, NGF, and substance P gene expression compared to IL‐1β alone. WIN‐55 alone increased NGF.
Animal studies				
Verrico et al. [34] 2020	Mice	Chemically induced (croton oil and LPS)	Topical (100 μL of 10 mg/mL CBD) and Intraperitoneal (1, 10, 100 μg CBD)	CBD reduced local and systemic inflammation by decreasing MPO activity, TNF‐α levels, and neutrophil influx. Additionally, CBD dose‐dependently reduce proinflammatory cytokines and increased anti‐inflammatory cytokines like IL‐10.
Philpott et al. [53] 2017	Rats	Chemically induced (MIA)	Topical (300 μg CBD)	CBD reduced leukocyte activity and synovial hyperemia.
Yimam et al. [57] 2021	Rats/Mice	Carrageenan‐induced paw edema model	Oral (5, 10, 20, 40 mg/kg CBD)	CBD dose‐dependently reduced inflammation and enhanced when combined with other drugs.
Carmon et al. [44] 2021	Mice	Surgically induced (DMM)	HU308 (0.5 μg in 10 μL, intra‐articular, 2×/week for 4 weeks)	HU308 reduced synovial inflammation and modulated macrophage/TLR signaling.
Burston et al. [43] 2013	Rats	Chemically induced (MIA)	Subcutaneous JWH133 (1 mg/kg)	JWH133 reduced proinflammatory cytokines (IL‐1b and TNFa) and increased anti‐inflammatory cytokine (IL‐10).
Analgesic effects				
Animal studies				
Rockel et al. [33] 2022	Mice	Surgical induced (DMM) Chemically induced (MIA)	DMM: THC (1, 5, 10 mg/kg) Intra‐articular (1×/week) Oral gavage (5×/week, 9 weeks) MIA: THC (5, 10 mg/kg) Oral gavage (5×/week, 3 weeks)	DMM: 10 mg/kg THC ↓ allodynia (week 9). MIA: 5, 10 mg/kg THC ↓ allodynia (Week 1), only 10 mg/kg at Week 3
Karuppagouder et al. [31] 2022	Mice	Surgical induced (DMM)	Topical CBD oil (50 mg/mL CBD, 20 mg/kg/day), CBG oil (25 mg/mL CBG + 25 mg/mL CBD, 10 mg/kg/day)	No effect on mechanical allodynia; significant reduction in cold allodynia (acetone test)
Philpott et al. [53] 2017	Rats	Chemically induced (MIA)	CBD (100–300 μg, local/topical)	300 μg CBD ↑ paw withdrawal threshold and weight bearing
Yimam et al. [57] 2021	Rats/Mice	Hot plate test	5% CBD, topical	↑paw withdrawal latency
Carmon et al. [44] 2021	Mice	Surgical induced (DMM)	HU308 (0.5 μg in 10 μL, intra‐articular, 2×/week for 4 weeks)	Reduced joint pain (limb withdrawal thresholds).
Yao et al. [56] 2008	Rats	Chemically induced (MIA)	A‐796260 (35 mg/kg, intraperitoneal)	A‐796260 reversed MIA‐induced grip force reduction, comparable to celecoxib.
Schuelert et al. [55] 2010	Rats	Chemically induced (MIA)	GW405833 (10^−6^ mol/100 μL, intra‐articular)	Significant shift in weight distribution, indicating increased pain response in the MIA joint
Burston et al. [43] 2013	Rats	Chemically induced (MIA)	Subcutaneous JWH133 (1 mg/kg, 1 mL/kg)	Attenuated decrease in weight‐bearing capacity; attenuated decrease in mechanical withdrawal thresholds in ipsilateral hind
Verrico et al. [34] 2020^a^	Dogs	Spontaneous OA	Oral: placebo, 20 mg/day naked CBD, 50 mg/day naked CBD, 20 mg/day liposomal CBD	Significant pain reduction with 50 mg/day naked or 20 mg/day liposomal CBD
Gamble et al. [48] 2018^a^	Dogs	Spontaneous OA	CBD or placebo, 2 mg/kg every 12 h	CBD significantly reduced pain at Weeks 2 and 4 vs. baseline
Brioschi et al. [42] 2020^a^	Dogs	Spontaneous OA	CBD oil vs Control, (2 mg/kg every 12 h)	CBD group showed significantly lower pain severity and interference at multiple time points.
Gabriele et al. [47] 2022^a^	Dogs	Spontaneous OA	14% cannabis sativa oil daily for 150 days	Significant pain reduction, more pronounced post physiotherapy
Mejia et al. [52] 2021^a^	Dogs	Spontaneous OA	2.5 mg/kg CBD oil every 12 h	No significant difference observed between CBD and placebo groups for pain severity/interference. Improvements observed within CBD group at Weeks 3 and 6 compared to baseline.

*Note:* ACAN, aggrecan; ADAMTs‐4, A disintegrin and metalloproteinase with thrombospondin motif 4; CBG, cannabigerol; CBD, cannabidiol; CBD‐PLGA‐NPs, cannabidiol‐poly (lactic‐co‐glycolic acid) nanoparticles; CCL2, C‐C motif chemokine ligand 2; COL2, collagen type II; D9‐THC, delta‐9‐tetrahydrocannabinol; DMSO, dimethyl sulfoxide; IL‐1β, interleukin‐1 beta; IL‐8, interleukin 8; LPS, lipopolysaccharide; MIA, monosodium iodoacetate; MPO, myeloperoxidase; MMP, matrix metalloproteinase; OA, osteoarthritis; THP‐1, human monocytic cell line; THC, tetrahydrocannabinol; TIMP, tissue inhibitor of metalloproteinases; TNFα, tumor necrosis factor alpha.

Abbreviations: ATCC, American Type Culture Collection; COMP, cartilage oligomeric matrix protein; DMM, destabilization of the medial meniscus; FLS, fibroblast‐like synoviocytes; mBMDM, murine bone marrow‐derived macrophages; NGF, nerve growth factor; PBMC, peripheral blood mononuclear cells; SEB, staphylococcal enterotoxin B; TKA, total knee arthroplasty; TKR, total knee replacement; TLR, Toll‐like receptor; TRPV1, transient receptor potential vanilloid 1.

^a^Randomized placebo‐controlled trial.

Furthermore, a study conducted in animals showed that administering THC orally can decrease cartilage degeneration [33]. However, intra‐articular administration of THC has been found to accelerate cartilage degeneration [33]. In one study, treatment with CBG ameliorated cartilage degeneration and chondrocyte loss by reducing the expression of the catabolic enzyme MMP13 in chondrocytes while promoting the production of the cartilage matrix [31]. Additionally, two studies reported that intra‐articular administration of CB2 receptor agonist (HU308 and JWH‐133) attenuated cartilage damage and osteophyte appearance and reduced the expression of MMP3, MMP9, and MMP13 in the cartilage [44, 51].

#### 3.1.3. Effect of Cannabis Extracts on Modulation of Proinflammatory Factors

Overall, nine studies (four using chondrocyte cells and five using OA animal models) reported the effect of cannabis extracts on the modulation of proinflammatory factors (Table 1 and Supporting Appendix 2) [34, 43–45, 49, 53, 54, 57]. The most common proinflammatory mediators studied included tumor necrosis factors (TNF‐α), IL‐1β, and IL‐6. Among these studies, five reported that CBD treatment resulted in a significant reduction in circulating TNF‐α, IL‐1β and IL‐6 levels [34, 45, 49, 54, 57], while other findings indicated that CBD treatment increased the level of anti‐inflammatory IL‐10 [34] and reduced myeloperoxidase (MPO) activity [34] and circulating neutrophils [53]. On the other hand, intra‐articular administration of THC increased synovitis in one study [33]. In studies that evaluated CB2 receptor agonists (HU308 and JWH133) on modulation of proinflammatory cytokines, HU308 reduced the expression of IL‐1β and IL‐6 and suppression of macrophage activity in the synovium [44, 54]. JWH133 reduced serum levels of TNF‐α and IL‐1β and increased that of IL‐10 [43].

#### 3.1.4. Effect of Cannabis Extracts on Pain

Thirteen preclinical studies were identified in which the analgesic effects of cannabis extracts were investigated in OA animal models and domestic dogs with OA (Table 1 and Supporting Appendix 2) [31, 33, 34, 42–44, 47, 48, 52, 53, 55–57]. The cannabis extracts examined included CBD, THC, and CB2 receptor agonists. Oral administration of THC was found to reduce mechanical allodynia, whereas intra‐articular administration failed to attenuate mechanical allodynia [33]. CBD reduced cold allodynia, increased hind‐paw withdrawal thresholds, improved hind limb weight bearing, and increased paw withdrawal latency [31, 53, 57]. The CB2 receptor agonist increased limb withdrawal thresholds and enhanced hindlimb grip force [43, 44, 56]. However, the CB2 receptor agonist GW405833 augmented the pain response in induced knee OA joints, as evidenced by increased hindlimb incapacitation [55]. CBD and *Cannabis sativa* supplementation significantly reduced pain in dogs diagnosed with OA, except in one study where there was no significant difference between CBD and placebo in pain reduction [34, 42, 47, 48, 52].

**FIGURE 2:**
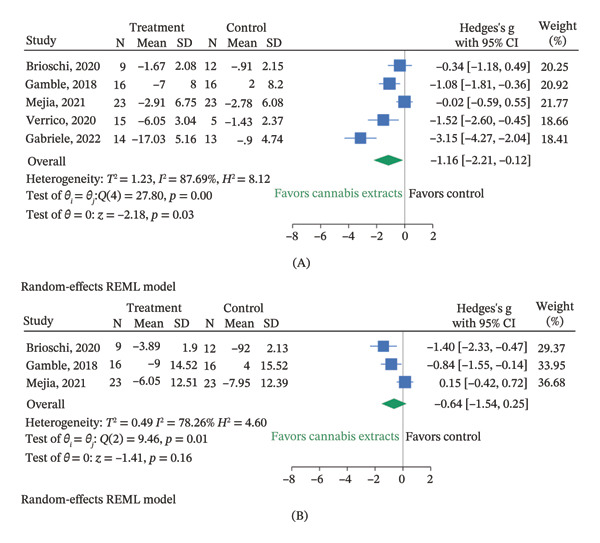
Forest plot showing the effects of cannabis extracts on (A) pain severity and (B) pain interference, measured using canine brief pain inventory (CBPI).

The meta‐analysis of five controlled studies was conducted separately by species and by outcome for studies that met the criteria for quantitative pooling. In dogs [34, 42, 47, 48, 52], the pooled effects of CBD indicated a reduction in pain severity (SMD: −1.16; 95% CI = −2.21–−0.12), while no ststistically significant reduction in pain interference (SMD: −0.65; 95% CI = −1.54–0.25) was observed. However, the certainty of the evidence remains low (Figure 2).

#### 3.1.5. Effect of Cannabis Extracts on Physical Function and QoL

Three studies used spontaneous OA models in domestic animals, while one used a surgically induced OA model in mice [31, 34, 42, 52]. Overall, the studies showed positive effects of CBD or CBG on mobility and QoL, although one study reported no significant difference between the CBD and placebo groups (Supporting Appendix 2).

No SAEs were reported in any of the studies included. However, mild AEs, such as increases in alkaline phosphatase (ALP), alanine aminotransferase (ALT), aspartate aminotransferase (AST), creatinine, and glucose levels over time, were observed. Other reported AEs in the included preclinical animal studies were vomiting, diarrhea, and somnolence (Supporting Appendix 2).

### 3.2. Clinical Studies

#### 3.2.1. Overall Description of Included Studies

Five RCTs and one open‐label trial, including a total of 606 patients investigated the effect of CBD [58–62] and THC [63] in human OA patients recruited from Denmark, Austria, USA, and Australia were included in this review (Supporting Appendix 2). Among these patients, 33% were female, 15% were male, and the sex of 52% of the participants was not reported. The mean age of the patients was 62.5 years. The duration of follow‐up ranged from 6 to 12 weeks. In all RCTs, the most evaluated outcome measures were analgesic efficacy and physical function, using validated questionnaires.

#### 3.2.2. Effect of Cannabis Extracts on OA Pain

All six studies assessed pain as a measure of analgesic efficacy, three in knee OA [60, 61, 63] and three in hand OA [58, 59, 62] using various patient‐reported outcomes such as the Western Ontario and McMaster Universities Osteoarthritis Index (WOMAC) pain subscale, visual analog scale (VAS), and numeric rating scale (NRS). Four RCTs evaluated pain intensity using VAS [59, 61–63], one trial used both WOMAC and VAS [61]. The remaining two trials assessed pain using WOMAC pain [60] and NRS [58].

**FIGURE 3:**
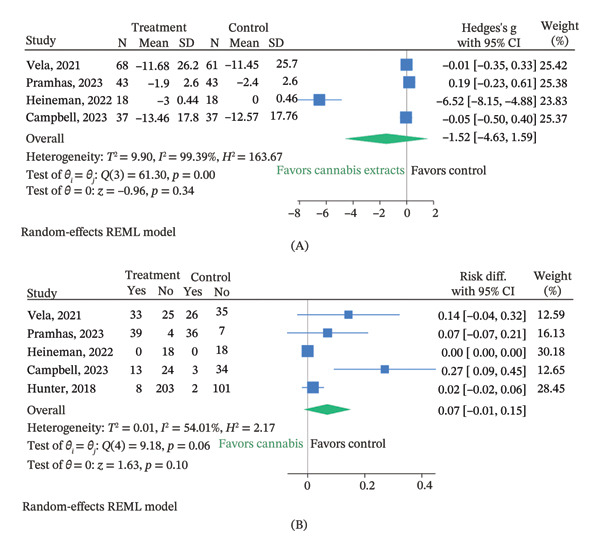
Forest plot showing the effects of cannabis extracts on (A) pain reduction and (B) adverse events in human studies.

One double‐blind RCT [62] examined oral CBD compared to placebo in patients with hand OA and psoriatic arthritis over a period of 12 weeks and found no statistically significant mean difference in pain intensity (VAS, 0–100 scale, MD 0.23, 95% CI −9.41–9.90) at 12 weeks. In addition, a reduction of pain intensity (≥ 30%; RR 1.01, 95% CI 0.66–1.55) was not statistically significant in the CBD group compared to the placebo group. On the other hand, two small cross‐over trials in patients with hand OA reported improvement in pain with CBD treatment compared to the control arm [58, 59]. Three RCTs investigated the effect of CBD on pain reduction in patients with knee OA [60, 61, 63]. The results indicated no statistically significant difference in pain reduction when compared to the placebo group. Meta‐analysis of four RCTs found no evidence of improvement in pain (SMD: −1.52; 95% CI = −4.63–1.59) (Figure 3).

#### 3.2.3. Effect of Cannabis Extracts on OA‐Related Physical Function

In three RCTs and one open‐label trial, the effects of CBD on functional limitations were investigated using various patient‐reported outcome measures, including the WOMAC function subscale, the Health Assessment Questionnaire Disability Index (HAQ‐DI), the Disabilities of the Arm, Shoulder, and Hand (DASH) questionnaire, and the Functional Index for Hand OA (FIHOA) [58, 59, 61, 62]. Across the three RCTs [59, 61, 62], comparisons of CBD with placebo revealed no significant improvements in functional limitations compared to placebo. Furthermore, the open‐label trial [58], conducted without a comparison group, found no improvement in self‐reported functionality.

#### 3.2.4. Effect of Cannabis Extracts on QoL

In one RCT [61] and an open‐label trial [58], QoL was assessed using the SF‐36 (Short Form Health Survey) and NRSs, respectively. The results of the RCT showed no significant improvement in the CBD group compared to the placebo [61]. However, in the open‐label trial, QoL showed improvement after CBD application [58].

### 3.3. Safety Outcome

Six studies, three in knee OA [60, 61, 63] and three in hand OA [58, 59, 62] reported AEs (Supporting Appendix 2). The most frequently reported AEs were associated with the gastrointestinal, the neurological, and the musculoskeletal AEs. A greater number of patients in the CBD treatment group reported AEs compared to those in the placebo group. However, these AEs were generally mild and well tolerated and did not result in any participants withdrawing from the studies. In the CBD groups, two patients reported severe AEs: One participant was diagnosed with ductal carcinoma, and another experienced lipothymia. Similarly, in the placebo groups, two severe AEs were reported: One participant suffered an acute shoulder fracture, and another developed malignant hypertension [62]. None of these severe AEs were deemed to be related to adverse drug reactions. Meta‐analyses of five studies indicated that the cannabis extracts group had 7% higher AEs (Figure 3).

### 3.4. Risk of Bias and Certainty of Evidence

The risk of bias assessment for preclinical and clinical studies is shown Supporting Appendix 2. In preclinical studies, the risk of selective outcome reporting was unclear because there was no information available on whether all the prespecified primary and secondary outcomes of interest of a study had been reported, and the study protocols were not available. Two of the six human studies had a high overall risk of bias due to missing outcome data, measurement of the outcome, and the randomization process [58, 60]. One of these studies was available only as a conference abstract, which further contributed to the lack of sufficient detail in these areas [60]. Three RCTs had “some concerns” of overall risk of bias, mainly because of the randomization process, missing outcome data, and selection of reported results [59, 62, 63].

The certainty of clinical evidence was low to moderate, and current evidence did not demonstrate consistent improvements in pain, function, or QoL compared with placebo or alternative comparison group assessed using the Grade of Recommendation, Assessment, Development and Evaluations(GRADE) (Supporting Appendix 2). Publication bias was not formally assessed because fewer than 10 studies were available for each quantitative synthesis, consistent with the Cochrane recommendations.

## 4. Discussion

To our knowledge, this is the first systematic review to synthesize both preclinical and clinical evidence on the efficacy and safety of cannabis‐based extracts in OA. The goal was to evaluate whether these compounds offer potential therapeutic benefits and to clarify the strength and consistency of supporting evidence across preclinical and clinical settings.

### 4.1. Preclinical Evidence

Across heterogeneous preclinical models, some evidence suggested that cannabis extracts exert anti‐inflammatory, chondroprotective, and analgesic effects relevant to OA. Several studies reported reductions in mechanical and thermal hyperalgesia [31, 33, 34, 42–44, 47, 48, 53, 56, 57], and in vitro studies indicated modulation of inflammatory cytokines such as IL‐1β, TNF‐α, and IL‐6, alongside increases in the anti‐inflammatory cytokine IL‐10 [34, 45, 49, 54, 57]. Other studies reported that cannabinoid exposure may influence cartilage metabolism, including reductions in catabolic enzymes such as MMPs and potential increases in anabolic markers [44, 46, 49, 50].

In addition to enzymatic modulation, cannabis extracts have been reported to reduce chondrocyte loss [31] and restore TIMPs and COMP, both of which are crucial for maintaining cartilage structure and preventing excessive matrix degradation [51]. In preclinical models, cannabis extracts also reduced osteophyte formation, a hallmark of OA progression, thereby potentially decreasing joint stiffness and improving mobility [44]. Furthermore, they demonstrated chondroprotective properties by attenuating cartilage degeneration [31, 33, 44] and synovial inflammation [33, 44]. These effects of cannabis extracts were route of administration dependent in some studies, with significant improvement in maintaining cartilage integrity and reduced synovitis with oral administration of THC compared to intra‐articular administration of THC [33].

Additionally, preclinical studies have shown that cannabis extracts improve gait and locomotor activity [31, 34]. These findings are consistent with the hypothesis that cannabinoid‐based therapies may influence mobility in animal models of OA,although confidence in these effects remains limited by substantial between‐study heterogeneity in cannabinoid formulation, route of administration, dose, OA phenotype, outcome assessment, and study design constraints.

These effects were, however, observed under heterogeneous conditions and should not be interpreted as conclusive findings across the literature. The preclinical literature is characterized by inconsistent OA induction methods, heterogeneous cannabinoid formulations, and frequent lack of randomization or blinding factors that collectively introduce substantial risk of bias and limit reproducibility. Few studies quantified dose–response relationships, examined pharmacokinetics, or employed standardized cannabinoid preparations. Accordingly, while these experimental findings provide biological plausibility for anti‐inflammatory, chondroprotective, and analgesic actions, the evidence remains preliminary and low certainty.

Mechanistic signals observed in preclinical models have not translated into demonstrated clinical efficacy, a gap that likely reflects species differences, endocannabinoid receptor distribution, formulation heterogeneity, and the limited quality of available trials. Moreover, many preclinical formulations used purified cannabinoids under controlled conditions, which differ markedly from the heterogeneous extracts used in clinical trials. Standardization of active components (THC and CBD) and exploration of pharmacokinetics in humans are essential to bridge this translational divide.

### 4.2. Clinical Evidence

Evidence from six clinical trials remains inconclusive. The two studies reporting modest benefits were limited by small sample size, lack of comparator groups, and a high risk of bias [58, 59]. The remaining RCTs found no significant differences in pain, function, or QoL compared with placebo, indicating low‐certainty evidence for clinical efficacy [60–63].

Current clinical evidence does not demonstrate consistent efficacy of cannabis extracts for pain, physical function, or QoL in OA, and the certainty of evidence remains low. The discordance between preclinical and clinical findings underscores the need for more rigorous, adequately powered RCTs employing standardized cannabinoid formulations and validated OA outcome measures. The null findings observed across RCTs are likely attributable to several methodological factors, including small sample sizes, short follow‐up durations of 6–12 weeks, heterogeneous cannabinoid formulations and dosing regimens, and the absence of standardized outcome measures, all of which limit the ability to detect a true treatment effect even if one exists.

### 4.3. Safety

Across clinical studies, cannabis‐based products were generally well tolerated, with AEs typically mild to moderate in severity. The most common were gastrointestinal (nausea, dry mouth), neurological (dizziness, somnolence), and musculoskeletal systems; few withdrawals were attributable to treatment [58–63]. Nevertheless, the limited sample sizes and short treatment durations preclude firm conclusions regarding long‐term safety.

### 4.4. Comparison with Previous Studies

There was limited evidence available to compare the findings of our review. Our results align with prior systematic reviews of cannabinoids for chronic noncancer pain and rheumatic diseases, which also concluded that analgesic effects are modest and evidence quality remains low [64–70]. In chronic musculoskeletal and neuropathic pain conditions, cannabinoids have demonstrated small improvements in pain, sleep quality, anxiety, and QoL compared to placebo [64, 71]. Similarly, previous animal reviews have described antinociceptive effects in persistent pain models [72] yet emphasized the lack of translational data. Together, these findings indicate that while cannabinoids have plausible biological activity, clinical benefit in OA has not been convincingly demonstrated. Regarding safety, the findings of our systematic review are in line with the findings of earlier literature reviews of cannabinoids in rheumatic disease [68, 69], and three systematic reviews of cannabinoids in patients with chronic pain [64, 66, 70] have similarly reported predominantly nonserious AEs, most often dose related and reversible upon treatment cessation.

### 4.5. Limitations

This review has some limitations. We only considered studies written in English, and two preclinical studies [32, 33] and one clinical study [60] were available only as conference abstracts without full‐text publications. Additional limitations include a lack of long‐term outcomes and AE reporting, small sample sizes, and limited exploration of the opioid‐sparing effects of cannabinoids. This review also did not assess the potential effects of cannabis extracts on mood, sleep quality, or depression, which are clinically important domains in chronic pain populations given the bidirectional relationship between pain and psychological wellbeing. Furthermore, our review focused exclusively on cannabis extracts and did not examine the potential contributions of noncannabinoid cannabis compounds, such as terpenes and flavonoids. Both of these areas warrant dedicated investigation in future trials and systematic reviews. Despite these limitations, this review adhered to a preregistered protocol, applied rigorous risk of bias assessment, and comprehensively searched multiple databases and registries, providing the most up‐to‐date synthesis of cannabinoid therapy in OA.

## 5. Conclusion

Preclinical evidence provideslow‐certainty preliminary support for anti‐inflammatory, analgesic, and chondroprotective effects of cannabinoid‐based therapies in OA models.However, current clinical evidence remains insufficient to establish efficacy for pain,function, or QoL. Large, adequately powered RCTs using standardized cannabinoid formulations are required to determine whether cannabis extracts provide clinically meaningful benefits in OA and to establish their long‐term safety profile.

## Author Contributions

Bedru J. Abafita: formal analysis, data curation, methodology, software, original drafting of the article, and review and editing; Benny Antony: conceptualization, data curation, critical revision of the article for important intellectual content, and final approval of the article; Dawn Aitken: data curation, critical revision of the article for important intellectual content, and final approval of the article; Ambrish Singh: data curation, methodology, critical revision of the article for important intellectual content, and final approval of the article.

## Funding

No funding was received for this manuscript. Open access publishing facilitated by University of Tasmania, as part of the Wiley—University of Tasmania agreement via the Council of Australasian University Librarians.

## Ethics Statement

This systematic review does not include any studies involving human or animal subjects conducted by any of the authors.

## Conflicts of Interest

The authors declare no conflicts of interest.

## Supporting Information

Additional supporting information can be found online in the Supporting Information section.

## Supporting information


**Supporting Information** Supporting information accompanying this manuscript include Supporting Appendix 1, which details the search strategy used to identify eligible studies, and Supporting Appendix 2, which includes additional figures and tables describing risk of bias assessments, characteristics of included preclinical and clinical studies, AEs, and certainty‐of‐evidence assessments. The PRISMA 2020 checklist is also provided to ensure transparent reporting in accordance with established guidelines.

## Data Availability

All data supporting the findings of this review are available within the article and its supporting information.
