# Supplementary material for: Efficacy and Safety of Cannabis Extracts for the Treatment of Osteoarthritis: A Systematic Review and Meta‐Analysis of Preclinical and Human Studies
Source: Pain Res Manag. 2026 Jul 27;2026:3998239. doi: 10.1155/prm/3998239 (PMC13408433; doi:10.1155/prm/3998239)
Supplement: Supplementary file 1 — Supporting Information Supporting information accompanying this manuscript include Supporting Appendix 1, which details the search strategy used to identify eligible studies, and Supporting Appendix 2, which includes additional figures and tables describing risk of bias assessments, characteristics of included preclinical and clinical studies, AEs, and certainty‐of‐evidence assessments. The PRISMA 2020 checklist is also provided to ensure transparent reporting in accordance with established guidelines. [file PRM-2026-3998239-s001.zip › Supplementary appendix 1.docx]

**Supplementary Material 1: Search Strategy**

**Appendix 1. EMBASE search strategy**

| 1. | osteoarthritis/ |
| --- | --- |
| 2. | exp osteoarthritis/ |
| 3. | 1 or 2 |
| 4. | exp cannabis/ |
| 5. | cannabis.mp. |
| 6. | exp cannabinoid/ |
| 7. | exp cannabinol/ |
| 8. | cannabinol.mp. |
| 9. | marijuana.mp. |
| 10. | exp cannabidiol/ |
| 11. | cannabidiol.mp. |
| 12. | phytocannabinoid.mp. |
| 13. | exp tetrahydrocannabinol/ |
| 14. | tetrahydrocannabinol.mp. |
| 15. | exp dronabinol/ |
| 16. | dronabinol.mp. |
| 17. | nabilone.mp. |
| 18. | sativex.mp. |
| 19. | cbd.mp. |
| 20. | thc.mp. |
| 21. | 4 or 5 or 6 or 7 or 8 or 9 or 10 or 11 or 12 or 13 or 14 or 15 or 16 or 17 or 18 or 19 or 20 |
| 22. | 3 and 21 |

**Appendix 2. CNAHL search strategy**

| **#** | **Query** | **Results** |
| --- | --- | --- |
| S21 | S6 AND S20 | 50 |
| S20 | S7 OR S8 OR S9 OR S10 OR S11 OR S12 OR S13 OR S14 OR S15 OR S16 OR S17 OR S18 OR S19 | 24,976 |
| S19 | (MM "Medical Marijuana") | 1,884 |
| S18 | TI THC OR AB THC | 1,546 |
| S17 | TI CBD OR AB CBD | 2,077 |
| S16 | TI sativex OR AB sativex | 64 |
| S15 | TI nabilone OR AB nabilone | 105 |
| S14 | TI dronabinol OR AB dronabinol | 151 |
| S13 | TI tetrahydrocannabinol OR AB tetrahydrocannabinol | 729 |
| S12 | TI phytocannabinoid OR AB phytocannabinoid | 197 |
| S11 | TI cannabidiol OR AB cannabidiol | 1,288 |
| S10 | TI cannabinol OR AB cannabinol | 65 |
| S9 | TI cannabinoid* OR AB cannabinoid* | 4,009 |
| S8 | TI marijuana OR AB marijuana | 8,537 |
| S7 | TI cannabis OR AB cannabis | 12,298 |
| S6 | S1 OR S2 OR S3 OR S4 OR S5 | 44,200 |
| S5 | (MM "Osteoarthritis+") | 26,395 |
| S4 | TI arthrosis OR AB arthrosis | 905 |
| S3 | TI degenerative arthritis OR AB degenerative arthritis | 451 |
| S2 | TI osteoarthr* OR AB osteoarthr* | 37,405 |
| S1 | TI Osteoarthritis OR AB Osteoarthritis | 35,619 |

**Appendix 3. MEDLINE search strategy**

| **#** | **Query** | **Results** |
| --- | --- | --- |
| S21 | S6 AND S20 | 682 |
| S20 | S7 OR S8 OR S9 OR S10 OR S11 OR S12 OR S13 OR S14 OR S15 OR S16 OR S17 OR S18 OR S19 | 88,655 |
| S19 | MM THC OR TX THC | 10,257 |
| S18 | MM CBD OR TX CBD | 12,953 |
| S17 | MM sativex OR TX sativex | 382 |
| S16 | MM nabilone OR TX nabilone | 530 |
| S15 | MM dronabinol OR TX dronabinol | 8,960 |
| S14 | MM tetrahydrocannabinol OR TX tetrahydrocannabinol | 5,690 |
| S13 | MM phytocannabinoid OR TX phytocannabinoid | 1,642 |
| S12 | MM cannabidiol OR TX cannabidiol | 7,837 |
| S11 | MM cannabinol OR TX cannabinol | 978 |
| S10 | MM cannabinoid* OR TX cannabinoid* | 35,768 |
| S9 | MM marijuana OR TX marijuana | 27,450 |
| S8 | MM cannabis OR TX cannabis | 39,267 |
| S7 | (MM "Medical Marijuana") | 2,163 |
| S6 | S1 OR S2 OR S3 OR S4 OR S5 | 169,169 |
| S5 | MM arthrosis OR TX arthrosis | 7,264 |
| S4 | MM degenerative arthritis OR TX degenerative arthritis | 2,441 |
| S3 | MM osteoarthr* OR TX osteoarthr* | 163,998 |
| S2 | MM Osteoarthritis OR TX Osteoarthritis | 156,535 |
| S1 | (MM "Osteoarthritis+") | 64,475 |

**Appendix 4. Cochrane library search strategy**

#1 MeSH descriptor: [Osteoarthritis] explode all trees 10843

#2 ("osteoarthritis"):ti,ab,kw OR (Osteoarthrosis):ti,ab,kw OR (Osteoarthroses):ti,ab,kw OR (Osteoarthritides):ti,ab,kw OR (Degenerative Arthritides):ti,ab,kw 24307

#3 #1 OR #2 24307

#4 MeSH descriptor: [Cannabis] explode all trees 641

#5 (cannabi* or Cannabis sativa or Marijuana or marihuana or ganja or bhang or hemp*):ti,ab,kw OR (cannabinoid*):ti,ab,kw OR (cannabidiol):ti,ab,kw OR (tetrahydrocannabinol):ti,ab,kw OR (dronabinol):ti,ab,kw 6146

#6 #4 or #5 6146

#7 #3 AND #6 42
