# Supplementary material for: Efficacy and Safety of Cannabis Extracts for the Treatment of Osteoarthritis: A Systematic Review and Meta‐Analysis of Preclinical and Human Studies
Source: Pain Res Manag. 2026 Jul 27;2026:3998239. doi: 10.1155/prm/3998239 (PMC13408433; doi:10.1155/prm/3998239)
Supplement: Supplementary file 1 — Supporting Information Supporting information accompanying this manuscript include Supporting Appendix 1, which details the search strategy used to identify eligible studies, and Supporting Appendix 2, which includes additional figures and tables describing risk of bias assessments, characteristics of included preclinical and clinical studies, AEs, and certainty‐of‐evidence assessments. The PRISMA 2020 checklist is also provided to ensure transparent reporting in accordance with established guidelines. [file PRM-2026-3998239-s001.zip › Supplementary appendix 2.docx]

Supplementary appendix 2

Supplement to “Efficacy and safety of cannabis extracts for the treatment of osteoarthritis: A systematic review of pre-clinical and human studies”

Contents

[**Figure 1**: Risk of bias assessment for the randomised controlled trials in humans included in this systematic review. 2](#_Toc190420438)

[**Table 1:** Characteristics of included pre-clinical studies for the effect of cannabis extracts on structural changes. 3](#_Toc190420439)

[**Table 2:** Characteristics of pre-clinical studies on the effects of cannabis extracts in modulating pro-inflammatory factors 4](#_Toc190420440)

[**Table 3:** Characteristics of included pre-clinical studies for the effect of cannabis extracts on pain. 5](#_Toc190420441)

[**Table 4:** Characteristics of included pre-clinical studies for the effect of cannabis extracts on physical function and gait analysis. 7](#_Toc190420442)

[**Table 5:** Adverse events observed in pre-clinical studies. 8](#_Toc190420443)

[**Table 6:** Key description of the included human studies. 9](#_Toc190420444)

[**Table 7:** Adverse events observed in clinical studies. 11](#_Toc190420445)

[**Table 8:** OHAT risk of bias table for pre-clinical studies 13](#_Toc190420446)

[**Table 9:** GRADEpro GDT certainty of evidence 14](#_Toc190420447)

[**Table 10**: OHAT risk of bias table for pre-clinical studies with reason for judgment 15](#_Toc190420448)

[**Table 11:** Risk of bias table for human studies 27](#_Toc190420449)

[Reference 29](#_Toc190420450)

**A**

**
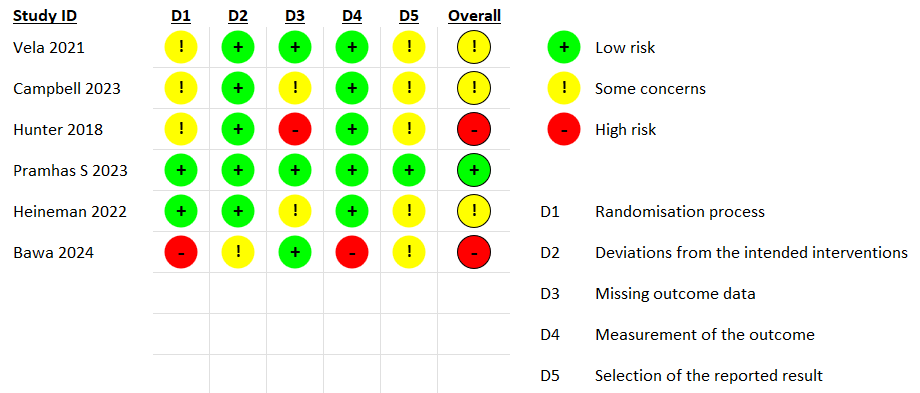
**

**B**

# **Figure 1**: Risk of bias assessment for the randomised controlled trials in humans included in this systematic review.

# **Table 1:** Characteristics of included pre-clinical studies for the effect of cannabis extracts on structural changes.

| **Animal studies** | | | | | |
| --- | --- | --- | --- | --- | --- |
| **Author** | **Animal model**  **Number**  **Gender**  **Age / weight** | **OA model** | **Administration route/dose** | **Start point and end point** | **Finding** |
| Rockel^1^ 2022 | C57BL/6 male mice (14-16 weeks) | Surgical induced (DMM) & chemically induced OA (MIA) | DMM: THC (1, 5 or 10 mg/kg) via intra-articular injection (1x/week) or oral (5x/week for 9 weeks)  MIA: THC (5 or 10mg/kg) via oral (5x/week for 3 weeks) | Start: 1-week post- DMM  End: 10 weeks post- DMM | Intra-articular THC accelerated cartilage degeneration and increased synovitis  Oral D9-THC reduced cartilage degeneration. |
| Karuppagouder^2^ 2022 | C57BL/6J male mice  N=6-8 per group  12-week-old | Surgical induced (DMM) | Topical CBD oil (20 mg/kg/day) or CBG oil (10 mg/kg/day) | Start: 3 days post DMM.  End: 8 weeks post DMM | CBG oil reduced cartilage degeneration, preserved chondrocytes, and reduced total volume of the subchondral bone |
| Carmon^3^ 2021 | Wild type  Male and female  3-month-old | Surgical induced (DMM) | HU308 (0.5 μg) intra-articular (2x/week for 4 weeks) | Start: 4 weeks post DMM  End: 8 weeks post DMM | IA administered HU308 attenuated cartilage damage and osteophyte appearance compared to mice treated with vehicle or left untreated. |
| Malek^4^ 2022 | Wistar rats  Male | Chemically induced OA MIA | JWH-133 was administered via intra-articular route at the dose of 100 ng per injection every 2^nd^ day for 2 weeks | Start: 14 days post MIA  End: 28 days post MIA | JWH-133 treatment restored the protein levels of TIMP1 and COMP in OA-affected cartilage and reduced the expression of MMPs such as MMP3, MMP9, and MMP13 in the cartilage. |
| Abbreviations. CBD – Cannabidiol; CBG – Cannabigerol; COMP – Cartilage Oligomeric Matrix Protein; D9-THC – Delta-9-Tetrahydrocannabinol; DMM – Destabilization of the Medial Meniscus; MIA – Monosodium Iodoacetate; MMPs – Matrix Metalloproteinases; TIMP – Tissue Inhibitor of Metalloproteinases | | | | | |

# **Table 2:** Characteristics of pre-clinical studies on the effects of cannabis extracts in modulating pro-inflammatory factors

| **Animal studies** | | | | | |
| --- | --- | --- | --- | --- | --- |
| **Author** | **Animal model**  **Number**  **Gender**  **Age / weight** | **OA model** | **Administration**  **(route/dose)** | **Start point and**  **end point** | **Finding** |
| Verrico^5^ 2020 | C57BL/6 J mic  6- to 10-week-old | Chemically induced (croton oil, LPS) | Topical (100 μl of 10 mg/ml CBD) and Intraperitoneal (1, 10, 100 μg CBD) | NA | CBD reduced local and systemic inflammation by decreasing MPO activity, TNF-α levels, and neutrophil influx. Additionally, CBD dose-dependently reduce pro-inflammatory cytokines and increased anti-inflammatory cytokines like IL-10. |
| Philpott^6^ 2017 | Wistar rats  Male  150-175 g | Chemically induced (MIA) | Topical (300 μg CBD) | Start: Immediately after MIA  End:1 day post-MIA | CBD reduced leukocyte activity and synovial hyperemia. |
| Yimam^7^ 2021 | Sprague Dawley rats and CD-1 mice  5/group  8-week-old | Carrageen-induced paw edema model | Oral (5, 10, 20, 40 mg/kg CBD) | Start: Immediately after carrageenan inoculation  End: NA | CBD dose-dependently reduced inflammation, enhanced when combined with other drugs. |
| Carmon^3^ 2021 | C57BL/6  Female  3-month-old | Surgically induced (DMM) | HU308 (0.5 μg in 10 μL, intra-articular, 2x/week for 4 weeks) | Start: 4 weeks post-DMM.  End: 8 weeks post-DMM | HU308 reduced synovial inflammation, modulated macrophage/TLR signaling. |
| Burston^8^ 2013 | Sprague Dawley rats  Adult male  (180–200 g) | Chemically induced (MIA) | Subcutaneous JWH133 (1 mg/kg) | Start: 7 days post-MIA  End: NA | JWH133 reduced pro-inflammatory cytokines (IL-1b and TNFa) and increased anti-inflammatory cytokine (IL-10). |
| Abbreviations. OA: Osteoarthritis; LPS: Lipopolysaccharide; MIA: Monosodium Iodoacetate; CBD: Cannabidiol; MPO: Myeloperoxidase; CB2: Cannabinoid Receptor Type 2; TRPV1: Transient Receptor Potential Vanilloid 1; DMM: Destabilization of the Medial Meniscus; IL-1β: Interleukin-1 Beta; TNFα: Tumor Necrosis Factor Alpha; TLR: Toll-like Receptor | | | | | |

# **Table 3:** Characteristics of included pre-clinical studies for the effect of cannabis extracts on pain.

| **Animal studies** | | | | | |
| --- | --- | --- | --- | --- | --- |
| **Author** | **Animal model**  **Number**  **Gender**  **Age/weight** | **OA model** | **Administration (route/dose)** | **Start point and End point** | **Finding** |
| Rockel^1^ 2022 | C57BL/6 mice male  14–16-week-old | Surgical induced (DMM)  Chemically induced (MIA) | DMM: THC (1, 5, 10 mg/kg)  Intra-articular (1x/weeks)  Oral gavage (5x/weeks, 9 weeks)  MIA: THC (5, 10 mg/kg)  Oral gavage (5x/weeks, 3 weeks) | Start: One-week post- DMM  End: 10 weeks post- DMM | DMM: 10 mg/kg THC ↓ allodynia (week 9).  MIA: 5, 10 mg/kg THC ↓ allodynia (week 1), only 10 mg/kg at week 3 |
| Karuppagouder^2^ 2022 | C57BL/6J male mice  (N=6-8, 12-week-old) | Surgical induced (DMM) | Topical CBD oil (50 mg/ml CBD, 20 mg/kg/day),  CBG oil (25 mg/ml CBG + 25 mg/ml CBD, 10 mg/kg/day) | Start: 3 days post DMM.  End: 8 weeks post DMM | No effect on mechanical allodynia.  Significant reduction in cold allodynia (acetone test). |
| Philpott^6^ 2017 | Wistar rats  (Male,  150-175 g) | Chemically induced (MIA) | CBD (100-300 µg, local/topical) | Start: Immediately after MIA  End: 14 days post-MIA | 300 µg CBD ↑ paw withdrawal threshold and weight-bearing |
| Yimam^7^ 2021 | Sprague Dawley rats, CD-1 mice (n=5, 8 weeks old) | Hot plate test | 5% CBD, topically on right hind paw | Start: Immediately after hot plate test  End: NA | ↑paw withdrawal latency |
| Carmon^3^ 2021 | Wild type (Male/Female, 3 months) | Surgical induced (DMM) | HU308 (0.5 μg in 10 μL, intra-articular, 2x/week for 4 weeks) | Start: 4 weeks post-DMM.  End: 8 weeks post-DMM | Reduced joint pain (limb withdrawal thresholds) |
| Yao^9^ 2008 | Sprague–Dawley rats (adult male, 250–300 g) | Chemically induced (MIA) | A-796260 (35 mg/kg, intraperitoneal) | Start; 20 days post MIA  End: NA | A-796260 reversed MIA-induced grip force reduction, comparable to celecoxib. |
| Schuelert^10^ 2010 | Wistar rats (Male, 250–450 g) | Chemically induced (MIA) | GW405833 (10^-6 mol/100 µl, intra-articular) | Start: 14 days post MIA  End: NA | Significant shift in weight distribution, indicating increased pain response in the MIA joint. |
| Burston^8^ 2013 | Sprague Dawley rats (adult male, 180–200g) | Chemically induced (MIA) | Subcutaneous JWH133 (1 mg/kg, 1 ml/kg) | Strat: 7 days post-MIA  End: 4 weeks post-MIA | - Attenuated decrease in weight-bearing capacity  - Attenuated decrease in mechanical withdrawal thresholds in ipsilateral hind |
| Verrico^5^ 2020 ^a^ | Dogs  N=20  41±15 kg | Spontaneous OA | Oral: Placebo, 20 mg/day naked CBD, 50 mg/day naked CBD, 20 mg/day liposomal CBD | Start: Baseline  End: 4 weeks | Significant pain reduction with 50 mg/day naked or 20 mg/day liposomal CBD |
| Gamble^11^ 2018 ^a^ | Dogs  N=22  10.4±3.3 year  27.0±7.7kg | Spontaneous OA | Placebo or CBD, 2 mg/kg every 12 hours | Start: Baseline  End: 4 weeks | CBD significantly reduced pain at weeks 2 and 4 vs. baseline |
| Brioschi^12^ 2020 ^a^ | Dogs  N=24  136.6±29.6 Month  28.9±12kg | Spontaneous OA | Control vs. CBD oil (2 mg/kg every 12 hours) | Start: Baseline  End: 12 weeks | CBD group showed significantly lower pain severity and interference at multiple time points (T1, T2, T3, and T4) |
| Gabriele^13^ 2022 ^a^ | Dogs  N=27  8-15 year  5-54 kg | Spontaneous OA | 0.2g/kg supplement (14% cannabis sativa oil, 9.6% Boswellia serrata, 0.8% ginger, 1.6% vitamin C) daily for 150 days | Start: Baseline  End: 170 days | Significant pain reduction, more pronounced post-physiotherapy |
| Mejia^14^ 2021 | Dogs  N=23  4 -14 year  22 - 63kg | Spontaneous OA | 2.5 mg/kg CBD oil every 12 hours | Start: Baseline  End: 16 weeks | No significant difference between CBD and placebo groups for pain severity/interference. Improvements observed within CBD group at weeks 3 and 6 compared to baseline. |
| ^a^ Randomised placebo-controlled trial  Abbreviations. CBD: Cannabidiol; DMM: Destabilization of the Medial Meniscus (a surgical model for osteoarthritis); MIA: Monoiodoacetate (a chemical model for inducing osteoarthritis); THC: Tetrahydrocannabinol; CBG: Cannabigerol; OA: Osteoarthritis; NA: Not avalaible | | | | | |

# **Table 4:** Characteristics of included pre-clinical studies for the effect of cannabis extracts on physical function and gait analysis.

| **Animal studies** | | | | | |
| --- | --- | --- | --- | --- | --- |
| **Author** | **Animal model**  **Number**  **Gender**  **Age/weight** | **OA model** | **Administration route/dose** | **Start point and End point** | **Finding** |
| Karuppagouder^2^ 2022 | C57BL/6J mice  N=6-8 per group  Male  12-week-old | Surgical induced (DMM) | CBD oil (20 mg/kg/day), CBG oil (10 mg/kg/day CBG + 10 mg/kg/day CBD), subcutaneous, every other day | Start: 3 days post DMM.  End: 8 weeks post DMM | CBD/CBG restored stride and stance length compared to vehicle treated at 2, 4, 6, and 8 weeks and improved sway distance at weeks 6 and 8 post-OA induction |
| Verrico^5^ 2020 * | Dogs  N=20  41±15 kg | Spontaneous OA | Oral: Placebo, 20 mg/day naked CBD, 50 mg/day naked CBD, 20 mg/day liposomal CBD | Start: Baseline  End: 4 weeks | Significant improvements in mobility (sitting, standing, walking, running). |
| Brioschi^12^ 2020 * | Dogs  N=24  136.6±29.6 Month  28.9±12kg | Spontaneous OA | Control vs. CBD oil (2 mg/kg every 12 hours) | Start: Baseline  End: 12 weeks | The quality of life was significantly higher in the CBD group one week after the treatment |
| Mejia^14^ 2021 * | Dogs  N=23  9.7±2.4 year  22 - 63kg | Spontaneous OA | 2.5 mg/kg CBD oil every 12 hours | Start: Baseline  End: 16 weeks | Improved %BWD when comparing different time points. However, there are no differences observed between the CBD and placebo groups |
| * Randomised placebo-controlled trial  Abbreviations. CBD: Cannabidiol; CBG: Cannabigerol; DMM: Destabilization of the Medial Meniscus (a surgical model for osteoarthritis); OA: Osteoarthritis; %BWD: Percentage of Body Weight Distribution | | | | | |

# **Table 5:** Adverse events observed in pre-clinical studies.

| **Animal studies** | | | | | |
| --- | --- | --- | --- | --- | --- |
| **Author, year** | **Type of RCT** | **Number of participants** | **Comparisons** | **Adverse events observed** | **Main finding** |
| Verrico^5^ 2020 | Parallel | 20(IG:15, CG:5) | CBD vs Placebo | Serum chemistry | No significant changes in CBC, metabolic panel, ALT/ALKP values. |
| Brioschi^12^ 2020 | Parallel | 21(IG:9, CG:12) | CBD vs MMA | Ptyalism  Somnolence  Ataxia | N%:2/9(22.2) vs 0/12(0)  N%:1/9(11.1) vs 2/12(16.7)  N%:1/9(11.1) vs 2/12(16.7) |
| Gamble^11^ 2018 | Crossover | 16(IG:16, CG:16) | CBD vs Placebo | Serum chemistry | No significant change: ALP, glucose, and creatinine increased over time from baseline within group. |
| Gabriele^13^ 2022 | Parallel | 27(IG:14, CG:13) | Cannabis sativa oil and Boswellia serrata vs placebo | NA | No adverse events |
| Mejia^14^ 2021 | Crossover | 23(IG:23, CG:23) | CBD vs Placebo | Withdrawal  Vomiting  ↑Serum ALP  ↑Serum ALT  ↑Serum AST | N%:1/23(4) vs 0/23(0)  N%:1/23(4) vs 0/23(0)  N%:14/23(60.9) vs 0/23(0)  N%:6/23(26) vs 0/23(0)  N%:3/23(13) vs 0/23(0) |
| Abbreviations. ALKP: Alkaline Phosphatase; ALT: Alanine Aminotransferase; AST: Aspartate Aminotransferase; CBD: Cannabidiol; CG: Control Group; IG: Intervention Group; MMA: Multimodal Analgesia | | | | | |

# **Table 6:** Key description of the included human studies.

| **Participants** | | | | | | | **Outcome** | | **Intervention** | |  | |
| --- | --- | --- | --- | --- | --- | --- | --- | --- | --- | --- | --- | --- |
| **Author (year)** | **Country, Trial Name, Registration No.** | **Study Design** | **Sample (IG, CG)** | **Age (Median/Mean)** | **Women (%)** | **Type of OA** | **Key outcomes** | **Assessment methods** | **Intervention groups** | **Control groups** | **Duration** | **Conclusion** |
| Vela^15^ 2021 | Denmark, NordCAN, NTC03693833 | RCT, double-blind, single center | 136 (70, 66) | IG:62, CG:61.5 | 64.7 | Hand OA & psoriatic arthritis | Pain intensity at 12 weeks | VAS | 20-30 mg CBD OD | Placebo | 12 weeks | No significant difference in pain reduction between groups. Both groups experienced a reduction in pain intensity from baseline, with no significant difference between the groups. |
| Pramhas^16^ 2023 | Austria, NCT04607603 | RCT,  double blind,  placebo  controlled, single center | 86 (43, 43) | IG:60, CG:65 | 69.8% | Knee OA | Knee pain  Function  Stiffness  Neuropathic pain | WOMAC,  VAS,  PainDETECT | CBD 200 mg TID | Placebo | 9 weeks | No significant differences in outcomes between groups. |
| Heineman^17^ 2022 | USA, NCT0461137 | RCT,  double blind,  placebo  controlled,  crossover, single center | 18 (18, 18) | 64.2±11 | 83.3 | Thumb basal joint arthritis | Thumb pain and  Functional improvement | VAS, DASH, SANE | 1ml of CBD cream BID | Control | 6 weeks | Significant improvement in pain and function in CBD group |
| Hunter^18^ 2018 | Australia, ACTRN12616001104448 | RCT, double-blind, single center | 314 (211,103) | 62 (41-78) | NA | Knee OA | Knee pain and physical function | WOMAC | 250 mg/500 mg transdermal CBD gel OD | Placebo | 12 weeks | No significant difference in pain, significantly more responders in the ZYN002 250 mg group compared to placebo. |
| Campbell^19^ 2023 | USA, NCT03098563 | Within-subject, double blind, randomised, placebo-controlled | 37 (37, 37) | 61.8 ± 6.7 | 64.9 | Knee OA | Clinical pain severity and physical function | VAS,  Performance based measuring physical function | 10 mg Dronabinol OD | Placebo | NA | No significant difference in pain and function. |
| Bawa^20^ 2024 | Australia, ACTRN12621001512819 | Open-label feasibility trial | 15(no comparator) | 60-69 | 73.3 | Hand OA | Hand pain, Grip strength, Function | NRS, FIHO | 0.25 ml CBD gel TID | NA | 5 weeks | Pain and grip strength improved over time following the application of transdermal CBD gel. |
| Abbreviation. BID: Twice daily; CBD: Cannabidiol; CG: Control Group; DASH: Disabilities of the Arm, Shoulder, and Hand (assessment); FIHO: Functional Index for Hand Osteoarthritis; IG: Intervention Group; NA: Not available; NRS: Numeric Rating Scale; OA: Osteoarthritis; OD: Once daily; RCT: Randomised Controlled Trial; SANE: Single Assessment Numeric Evaluation; TID: Three times daily; VAS: Visual Analog Scale; WOMAC: Western Ontario and McMaster Universities Osteoarthritis Index | | | | | | | | | | | | |

# **Table 7:** Adverse events observed in clinical studies.

| **Human studies** | | | | | |  |
| --- | --- | --- | --- | --- | --- | --- |
| **Author, year** | **Study design** | **Participants**  **(IG/CG)** | **Comparisons** | **Adverse events observed** | **Number AEs** | **Main finding** |
| Vela^15^ 2021 | RCT | 119 (58, 61) | CBD vs placebo | At least one AEs | 33/68(48.5) vs 26/61(42.6) | Higher gastrointestinal and urological AEs in placebo group |
|  |  |  |  | Total number of AEs | 58 vs 61 |  |
|  |  |  |  | SAEs | N%:2/58(3.4) vs 2/61(3.3) |  |
|  |  |  |  | Musculoskeletal | N%:11/58(19) vs 11/61(18) |  |
|  |  |  |  | Gastrointestinal system | N%:8/58(13.8) vs 19/61(31.1) |  |
|  |  |  |  | Neurological system | N%:4/58(6.9) vs 3/61(4.9) |  |
|  |  |  |  | Urological system | N%:0/58(0) vs 5/61(8.2) |  |
|  |  |  |  | Ear–nose–throat | N%:8/58(13.8) vs 0/61(0) |  |
|  |  |  |  | Dermatological system | N%:3/58(5.2) vs 0/61(0) |  |
|  |  |  |  | Cardiovascular | N%:4/58(6.9) vs 4/61(6.5) |  |
| Pramhas^16^ 2023 | RCT | 86 (43, 43) | CBD vs placebo | At least one AEs | 39/43(90.7) vs 36/43(83.7) | Higher AE frequency in CBD group |
|  |  |  |  | Total number of AEs | 135 vs 105 |  |
|  |  |  |  | Gastrointestinal system | 73 vs 58 |  |
|  |  |  |  | Hepatic system | 15 vs 5 |  |
|  |  |  |  | Neurological system | 30 vs 25 |  |
|  |  |  |  | Musculoskeletal system | 5 vs 4 |  |
|  |  |  |  | Cardiovascular system | 3 vs 2 |  |
|  |  |  |  | Dermatology and endocrine system | 4 vs 4 |  |
|  |  |  |  | Respiratory system | 3 vs 4 |  |
|  |  |  |  | Ophthalmological system | 2 vs 1 |  |
|  |  |  |  | Urological system | 2 vs 1 |  |
| Heineman^17^ 2022 | RCT | 18(18, 18) | CBD vs placebo | NA | - | No reported adverse events |
| Hunter^18^ 2018 | RCT | 314(211, 103) | CBD vs placebo | Application site dryness | N%:8/211(3.8) vs 1/103(0.9) | Mild application site and headache AEs in CBD group |
|  |  |  |  | Headache | N%:7/211(3.3) vs 2/103(1.9) |  |
| Campbell^19^ 2023 | RCT | 37(37, 37) | Dronabinol vs placebo | Total number of adverse events (mild and moderate) | N%:13/37(4.8) vs 3/37(1) | Higher AE frequency in dronabinol (THC) group |
| Bawa^20^ 2024 | Feasibility trial | 15 (IG only) | CBD | Total number of AEs | 32 | Multiple system AEs noted in CBD |
|  |  |  |  | Musculoskeletal system | 15 |  |
|  |  |  |  | Neurological system | 8 |  |
|  |  |  |  | Gastrointestinal system | 4 |  |
|  |  |  |  | Immunological system | 4 |  |
|  |  |  |  | Tooth extraction pain | 1 |  |
| In the studies by *Sibylle Pramhas et al. (2023)* and *Bawa Z et al. (2024)*, only the number of adverse events was reported, not the number of individuals who developed adverse events.  Abbreviation. AE: Adverse Event; CBD: Cannabidiol; CG: Control Group; IG: Intervention Group; NA: Not Applicable; RCT: Randomized Controlled Trial; SAE: Serious Adverse Event; THC: Tetrahydrocannabinol | | | | | | |

# **Table 8:** OHAT risk of bias table for pre-clinical studies


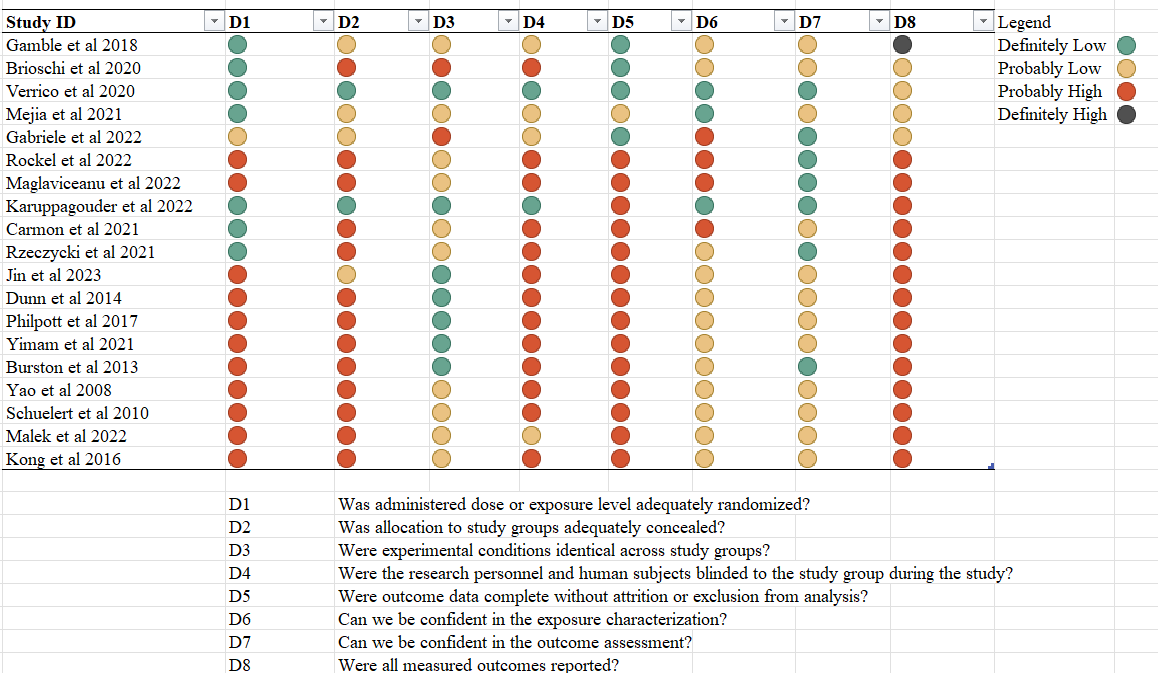


| **Table 9:** GRADEpro GDT certainty of evidence | | | | | | |
| --- | --- | --- | --- | --- | --- | --- |
| Outcomes | **Anticipated absolute effects^*^** (95% CI) | | Relative effect (95% CI) | № of participants (studies) | Certainty of the evidence (GRADE) | Comments |
|  | **Risk with Placebo** | **Risk with Cannabis extracts** |  |  |  |  |
| Pain (Pain) assessed with: VAS, WOMAC follow-up: range 6 weeks to 12 weeks | - | SMD **0.15 SD lower** (0.43 lower to 0.12 higher) | - | 315 (4 RCTs) | ⨁⨁◯◯ Low^a,b,c,d^ | Cannabis extracts probably does not reduce pain. |
| Function assessed with: WOMAC, HAQ-DI, DASH, FIHOA follow-up: range 6 weeks to 12 weeks | The effects of CBD on functional limitations were assessed using various patient-reported outcome measures, but no significant improvements were observed | |  | (4 RCTs) | ⨁⨁⨁◯ Moderate^a^ | Cannabis extracts are likely to result in minimal to no difference in the improvement of self-reported physical function |
| Quality of Life (QoL) assessed with: SF-36, NRS follow-up: range 6 weeks to 12 weeks | The results showed no significant improvement in the CBD group compared to the placebo. However, quality of life improved after CBD application in one of the trials | |  | (2 RCTs) | ⨁⨁◯◯ Low^e,f^ | The evidence suggests that cannabis extracts results in little to no difference in quality of Life. |
| Adverse Events  follow-up: range 6 weeks to 12 weeks | 256 per 1,000 | **18 per 1,000** (-3 to 38) | **RR 0.07** (-0.01 to 0.15)^g^ | 629 (5 RCTs) | ⨁⨁⨁◯ Moderate^b,h,i^ | Cannabis extracts may result in little to no difference in adverse events. |
| ***The risk in the intervention group** (and its 95% confidence interval) is based on the assumed risk in the comparison group and the **relative effect** of the intervention (and its 95% CI).  **CI:** confidence interval; **RR:** risk ratio; **SMD:** standardised mean difference | | | | | | |
| **GRADE Working Group grades of evidence** **High certainty:** we are very confident that the true effect lies close to that of the estimate of the effect. **Moderate certainty:** we are moderately confident in the effect estimate: the true effect is likely to be close to the estimate of the effect, but there is a possibility that it is substantially different. **Low certainty:** our confidence in the effect estimate is limited: the true effect may be substantially different from the estimate of the effect. **Very low certainty:** we have very little confidence in the effect estimate: the true effect is likely to be substantially different from the estimate of effect. | | | | | | |

a. There is some concern regarding risk of bias due to issues with randomization methods and the absence of study protocols, which suggests the possibility of selective reporting

b. Serious inconsistency. Numerous cannabis extracts and doses were evaluated across a relatively small number of trials, limiting the ability to draw clear conclusions regarding the efficacy and safety of the various cannabis extracts and doses administered.

c. All studies assessed VAS and WOMAC, which are validated measures of pain and important PROMs. Although the scales used varied between studies, they were standardized for the analysis.

d. Serious imprecision. The 95% confidence interval includes appreciable benefits based on effect size.

e. High risk of bias. One of the two trials had issues with randomization, lacked a comparison group, and participants were aware of the intervention they received

f. Serious inconsistency. A small number of trials, limiting our ability to draw conclusions.

g. Risk differences (RDs) are calculated from the pooled absolute difference in the risk of an event occurring between the intervention and control groups.

h. There is some concern regarding the risk of bias. Out of the five trials, one did not report adverse events, suggesting potential selective reporting bias.

i. Imprecision due to few events and confidence intervals include appreciable benefit or harm.

# **Table 10**: OHAT risk of bias table for pre-clinical studies with reason for judgment

| Risk of bias | Author judgment | Reason for judgment |
| --- | --- | --- |
| Gamble et al. (2018) |  |  |
| D1: Was administered dose or exposure level adequately randomized? | Definitely Low risk of bias | Animals were allocated to any study group including controls using a method with a random component |
| D2: Was allocation to study groups adequately concealed? | Probably Low risk of bias | There is indirect evidence that at the time of assigning study groups the research personnel did  not know what group animals were allocated |
| D3: Were experimental conditions identical across study groups? | Probably Low risk of bias | The same vehicle was used in control and experimental animals |
| D4: Were the research personnel and human subjects blinded to the study group during the study? | Probably Low risk of bias | There is indirect evidence that the research personnel were adequately blinded to study group |
| D5: Were outcome data complete without attrition or exclusion from analysis? | Definitely Low risk of bias | There is direct evidence that the loss of animals was adequately addressed, and reasons were documented. |
| D6: Can we be confident in the exposure characterization? | Probably Low risk of bias | Based on available information the study used validated methods for exposure assessment. However, analytical confirmation of CBD oil purity is explicitly stated. |
| D7: Can we be confident in the outcome assessment? | Probably Low risk of bias | There is indirect evidence that the assessors were adequately blinded to the study group |
| D8: Were all measured outcomes reported? | Probably High risk of bias | No protocol available |
| Brioschi et al. (2020) |  |  |
| D1: Was administered dose or exposure level adequately randomized? | Definitely Low risk of bias | Randomly assigned by computer |
| D2: Was allocation to study groups adequately concealed? | Probably High risk of bias | There is insufficient information provided about allocation to study groups |
| D3: Were experimental conditions identical across study groups? | Probably High risk of bias | There is indirect evidence that the vehicle differed between control and experimental animals |
| D4: Were the research personnel and human subjects blinded to the study group during the study? | Probably High risk of bias | There is indirect evidence that the research personnel were not adequately blinded to study group |
| D5: Were outcome data complete without attrition or exclusion from analysis? | Definitely Low risk of bias | There is direct evidence that the loss of animals was adequately addressed, and reasons were documented. |
| D6: Can we be confident in the exposure characterization? | Probably Low risk of bias | There is evidence that the exposure was independently characterized and consistently administered across treatment groups. |
| D7: Can we be confident in the outcome assessment? | Probably Low risk of bias | The outcome was assessed using well-established methods |
| D8: Were all measured outcomes reported? | Probably Low risk of bias | No protocol available |
| Verrico et al. (2020) |  |  |
| D1: Was administered dose or exposure level adequately randomized? | Definitely Low risk of bias | Study subjects were allocated to study group using a method with a random component. |
| D2: Was allocation to study groups adequately concealed? | Definitely Low risk of bias | At the time of recruitment, the research personnel and subjects did not know what study group subjects were allocated to. |
| D3: Were experimental conditions identical across study groups? | Definitely Low risk of bias | There is evidence that same vehicle was used in control and experimental animals. |
| D4: Were the research personnel and human subjects blinded to the study group during the study? | Definitely Low risk of bias | Research personnel were blinded to study group. |
| D5: Were outcome data complete without attrition or exclusion from analysis? | Definitely Low risk of bias | There is evidence that the loss of animals was adequately addressed, and reasons were documented. |
| D6: Can we be confident in the exposure characterization? | Definitely Low risk of bias | There is evidence that the exposure (including purity and stability of test substance) was independently characterized and consistently administered. |
| D7: Can we be confident in the outcome assessment? | Definitely Low risk of bias | The outcome was assessed using well-established methods and the outcome assessors were adequately blinded to the study group. |
| D8: Were all measured outcomes reported? | Probably Low risk of bias | No protocol available |
| Mejia et al. 2021 |  |  |
| D1: Was administered dose or exposure level adequately randomized? | Definitely Low risk of bias | Study subjects were allocated to study group using a method with a random component. |
| D2: Was allocation to study groups adequately concealed? | Probably Low risk of bias | There is indirect evidence that at the time of assigning study groups the research personnel did not know what group animals were allocated to. |
| D3: Were experimental conditions identical across study groups? | Probably Low risk of bias | There is indirect evidence that same vehicle was used in control and experimental animals. |
| D4: Were the research personnel and human subjects blinded to the study group during the study? | Probably Low risk of bias | There is indirect evidence that the research personnel were blinded to study group. |
| D5: Were outcome data complete without attrition or exclusion from analysis? | Probably Low risk of bias | There is direct evidence that the loss of animals was adequately addressed, and reasons were documented. |
| D6: Can we be confident in the exposure characterization? | Definitely Low risk of bias | There is direct evidence that the exposure was independently characterized, and purity confirmed, and exposure was consistently administered across treatment groups. |
| D7: Can we be confident in the outcome assessment? | Probably Low risk of bias | The outcome was assessed using acceptable methods and there is indirect evidence that the outcome assessors were blinded to the study group |
| D8: Were all measured outcomes reported? | Probably Low risk of bias | No protocol available |
| Gabriele et al. 2022 |  |  |
| D1: Was administered dose or exposure level adequately randomized? | Probably Low risk of bias | No details reported on sequence generation |
| D2: Was allocation to study groups adequately concealed? | Probably Low risk of bias | There is indirect evidence that at the time of assigning study groups the research personnel did not know what group animals were allocated to. |
| D3: Were experimental conditions identical across study groups? | Probably High risk of bias | There is indirect evidence that the vehicle differed between control and experimental animals |
| D4: Were the research personnel and human subjects blinded to the study group during the study? | Probably Low risk of bias | There is indirect evidence that the research personnel were blinded to study group |
| D5: Were outcome data complete without attrition or exclusion from analysis? | Definitely Low risk of bias | There is direct evidence that the loss of animals was adequately addressed, and reasons were documented. |
| D6: Can we be confident in the exposure characterization? | Probably High risk of bias | there is insufficient information provided about the validity of the exposure assessment method. |
| D7: Can we be confident in the outcome assessment? | Definitely Low risk of bias | There is direct evidence that the outcome was assessed using well-established methods |
| D8: Were all measured outcomes reported? | Probably Low risk of bias | No protocol available |
| Rockel et al., 2022* |  |  |
| D1: Was administered dose or exposure level adequately randomized? | Probably High risk of bias | There is insufficient information (randomization is not reported) |
| D2: Was allocation to study groups adequately concealed? | Probably High risk of bias | There is insufficient information provided about allocation to study groups |
| D3: Were experimental conditions identical across study groups? | Probably Low risk of bias | There is indirect evidence that the same vehicle was used in control and experimental animals |
| D4: Were the research personnel and human subjects blinded to the study group during the study? | Probably High risk of bias | There is insufficient information provided about blinding to study group during the study |
| D5: Were outcome data complete without attrition or exclusion from analysis? | Probably High risk of bias | There is insufficient information provided about loss of animals |
| D6: Can we be confident in the exposure characterization? | Probably High risk of bias | There is insufficient information provided about the validity of the exposure assessment method, but no evidence for concern |
| D7: Can we be confident in the outcome assessment? | Definitely Low risk of bias | The outcome was assessed using well-established methods, assessed at the same length of time after initial exposure in all study groups |
| D8: Were all measured outcomes reported? | Probably High risk of bias | No protocol available |
| Maglaviceanu et al., (2022) * |  |  |
| D1: Was administered dose or exposure level adequately randomized? | Probably High risk of bias | There is insufficient information (randomization is not reported) |
| D2: Was allocation to study groups adequately concealed? | Probably High risk of bias | There is insufficient information provided about allocation to study groups |
| D3: Were experimental conditions identical across study groups? | Probably Low risk of bias | There is indirect evidence that the same vehicle was used in control and experimental animals |
| D4: Were the research personnel and human subjects blinded to the study group during the study? | Probably High risk of bias | There is insufficient information provided about blinding to study group during the study |
| D5: Were outcome data complete without attrition or exclusion from analysis? | Probably High risk of bias | There is insufficient information provided about loss of study sample |
| D6: Can we be confident in the exposure characterization? | Probably High risk of bias | there is insufficient information provided about the validity of the exposure assessment method, but no evidence for concern |
| D7: Can we be confident in the outcome assessment? | Definitely Low risk of bias | The outcome was assessed using well-established methods, assessed at the same length of time after initial exposure in all study groups |
| D8: Were all measured outcomes reported? | Probably High risk of bias | No protocol available |
| Karuppagouder et al., (2022) |  |  |
| D1: Was administered dose or exposure level adequately randomized? | Definitely Low risk of bias | There is direct evidence that animals were allocated to any study group including controls using a method with a random component |
| D2: Was allocation to study groups adequately concealed? | Definitely Low risk of bias | There is direct evidence that at the time of assigning study groups the research personnel did not know what group animals were allocated to |
| D3: Were experimental conditions identical across study groups? | Definitely Low risk of bias | The same vehicle was used in control and experimental animals |
| D4: Were the research personnel and human subjects blinded to the study group during the study? | Definitely Low risk of bias | The research personnel were adequately blinded to study group |
| D5: Were outcome data complete without attrition or exclusion from analysis? | Probably High risk of bias | There is insufficient information provided about loss of animals beyond the adjustments made for anticipated attrition. |
| D6: Can we be confident in the exposure characterization? | Definitely Low risk of bias | There is direct evidence that the exposure (including purity and stability of the test substance) was independently characterized. |
| D7: Can we be confident in the outcome assessment? | Definitely Low risk of bias | The outcome was assessed using well-established methods and the outcome assessors were blinded to the study group. |
| D8: Were all measured outcomes reported? | Probably High risk of bias | No protocol available |
| Carmon et al., (2021) |  |  |
| D1: Was administered dose or exposure level adequately randomized? | Definitely High risk of bias | There is direct evidence that animals were allocated to study groups using a non-random method |
| D2: Was allocation to study groups adequately concealed? | Probably High risk of bias | There is insufficient information provided about allocation to study groups |
| D3: Were experimental conditions identical across study groups? | Probably Low risk of bias | There is indirect evidence that the same vehicle was used in control and experimental animals |
| D4: Were the research personnel and human subjects blinded to the study group during the study? | Probably High risk of bias | there is insufficient information provided about blinding to study group during the study |
| D5: Were outcome data complete without attrition or exclusion from analysis? | Probably High risk of bias | There is insufficient information provided about numbers of subjects lost to follow-up |
| D6: Can we be confident in the exposure characterization? | Probably High risk of bias | There is insufficient information provided about the validity of the exposure assessment method |
| D7: Can we be confident in the outcome assessment? | Probably Low risk of bias | The outcome was assessed using acceptable methods and assessed at the same length of time after initial exposure in all study groups |
| D8: Were all measured outcomes reported? | Probably High risk of bias | No protocol available |
| Rzeczycki et al., (2021) |  |  |
| D1: Was administered dose or exposure level adequately randomized? | Definitely Low risk of bias | Mice were allocated to any study group using a method with a random component. |
| D2: Was allocation to study groups adequately concealed? | Probably High risk of bias | There is insufficient information provided about allocation to study groups |
| D3: Were experimental conditions identical across study groups? | Probably Low risk of bias | The same vehicle was used in control and experimental animals. Housing conditions was identical across control and experimental groups |
| D4: Were the research personnel and human subjects blinded to the study group during the study? | Probably High risk of bias | There is insufficient information provided about blinding to study group during the study |
| D5: Were outcome data complete without attrition or exclusion from analysis? | Probably High risk of bias | There is insufficient information provided about numbers of subjects lost to follow-up |
| D6: Can we be confident in the exposure characterization? | Probably Low risk of bias | Because it uses established methods and has indirect evidence of consistent application of exposure protocols. However, the lack of mention of independent verification of purity for any chemicals used and potential lack of blinding in outcome assessment |
| D7: Can we be confident in the outcome assessment? | Definitely Low Risk of Bias | The study used validated methods and blinded histological assessments. |
| D8: Were all measured outcomes reported? | Probably High risk of bias | There is insufficient information provided about selective outcome reporting |
| Jin et al., (2023) |  |  |
| D1: Was administered dose or exposure level adequately randomized? | Probably High risk of bias | There is insufficient information provided about how subjects were allocated to study groups. The lack of explicit randomization is not uncommon in such settings, as the primary goal is often to establish dose-effect relationships rather than to evaluate the impact of random dosing |
| D2: Was allocation to study groups adequately concealed? | Probably Low risk of bias | The study did not involve human or animal subjects where selection bias might influence outcomes. t is deemed that lack of adequate allocation concealment would not appreciably bias results |
| D3: Were experimental conditions identical across study groups? | Definitely Low risk of bias | The study appears to have maintained identical experimental conditions across study groups |
| D4: Were the research personnel and human subjects blinded to the study group during the study? | Probably High risk of bias | It is unclear whether researchers conducting experiments and assessments were blinded to the treatment groups |
| D5: Were outcome data complete without attrition or exclusion from analysis? | Probably High risk of bias | There is no explicit indication of incomplete outcome data, attrition, or selective exclusion from analysis. |
| D6: Can we be confident in the exposure characterization? | Probably Low risk of bias | The study provides a comprehensive exposure characterization of CBD-PLGA-NPs through rigorous experimental methodologies and analytical techniques. There is indirect evidence that the exposure was independently characterized, and purity confirmed |
| D7: Can we be confident in the outcome assessment? | Probably Low risk of bias | The outcome was assessed using acceptable methods and assessed at the same length of time after initial exposure in all study groups |
| D8: Were all measured outcomes reported? | Probably High risk of bias | There is insufficient information provided about selective outcome reporting |
| Dunn et al., (2014) |  |  |
| D1: Was administered dose or exposure level adequately randomized? | Probably High risk of bias | There is insufficient information provided about randomization, however, the study is an in vitro experiment using cell cultures derived from human tissue. |
| D2: Was allocation to study groups adequately concealed? | Probably High risk of bias | There is insufficient information provided about allocation to study groups. It may not have been applicable to the study design. |
| D3: Were experimental conditions identical across study groups? | Definitely Low Risk of Bias | Identical experimental conditions across study groups. The study explicitly provides evidence that non-treatment-related experimental conditions and vehicle use were consistent |
| D4: Were the research personnel and human subjects blinded to the study group during the study? | Probably High risk of bias | The study does not provide clear information on whether blinding was implemented for research personnel |
| D5: Were outcome data complete without attrition or exclusion from analysis? | Probably High risk of bias | Not reported |
| D6: Can we be confident in the exposure characterization? | Probably Low risk of bias | Exposure was independently characterized and consistently administered |
| D7: Can we be confident in the outcome assessment? | Probably Low risk of bias | The outcome was assessed using well-established methods. However, there is no information that the outcome assessors were adequately blinded to the study group |
| D8: Were all measured outcomes reported? | Probably High risk of bias | There is insufficient information provided about selective outcome reporting |
| Philpott H et al., (2017) |  |  |
| D1: Was administered dose or exposure level adequately randomized? | Probably High risk of bias | There is insufficient information provided about how subjects were allocated to study groups |
| D2: Was allocation to study groups adequately concealed? | Probably High risk of bias | there is insufficient information provided about allocation to study groups |
| D3: Were experimental conditions identical across study groups? | Definitely Low risk of bias | The study appears to have maintained identical experimental conditions across study groups |
| D4: Were the research personnel and human subjects blinded to the study group during the study? | Probably High risk of bias | The study does not provide clear information on whether blinding was implemented for research personnel |
| D5: Were outcome data complete without attrition or exclusion from analysis? | Probably High risk of bias | Not reported |
| D6: Can we be confident in the exposure characterization? | Probably Low risk of bias | There is evidence that exposure was consistently administered across treatment groups, however, purity of chemical is not reported. |
| D7: Can we be confident in the outcome assessment? | Probably Low risk of bias | The outcome was assessed using well-established methods. However, there is no information that the outcome assessors were adequately blinded to the study group |
| D8: Were all measured outcomes reported? | Probably High risk of bias | There is insufficient information provided about selective outcome reporting |
| Yimam et al., 2021 |  |  |
| D1: Was administered dose or exposure level adequately randomized? | Probably High risk of bias | There is insufficient information provided about how subjects were allocated to study groups |
| D2: Was allocation to study groups adequately concealed? | Probably High risk of bias | there is insufficient information provided about allocation to study groups |
| D3: Were experimental conditions identical across study groups? | Definitely Low risk of bias | The study appears to have maintained identical experimental conditions across study groups |
| D4: Were the research personnel and human subjects blinded to the study group during the study? | Probably High risk of bias | The study does not provide clear information on whether blinding was implemented for research personnel |
| D5: Were outcome data complete without attrition or exclusion from analysis? | Probably High risk of bias | Not reported |
| D6: Can we be confident in the exposure characterization? | Probably Low risk of bias | There is evidence that exposure was consistently administered across treatment groups, however, purity of chemical is not reported. |
| D7: Can we be confident in the outcome assessment? | Probably Low risk of bias | The outcome was assessed using well-established methods. However, there is no information that the outcome assessors were adequately blinded to the study group |
| D8: Were all measured outcomes reported? | Probably High risk of bias | There is insufficient information provided about selective outcome reporting |
| Burston et al., 2013 |  |  |
| D1: Was administered dose or exposure level adequately randomized? | Probably High risk of bias | There is insufficient information provided about how subjects were allocated to study groups |
| D2: Was allocation to study groups adequately concealed? | Probably High risk of bias | There is insufficient information provided about allocation to study groups |
| D3: Were experimental conditions identical across study groups? | Definitely Low risk of bias | The study appears to have maintained identical experimental conditions across study groups |
| D4: Were the research personnel and human subjects blinded to the study group during the study? | Probably High risk of bias | The study does not provide clear information on whether blinding was implemented for research personnel |
| D5: Were outcome data complete without attrition or exclusion from analysis? | Probably High risk of bias | Not reported |
| D6: Can we be confident in the exposure characterization? | Probably Low risk of bias | There is evidence that exposure was consistently administered across treatment groups, however, purity of chemical is not reported. |
| D7: Can we be confident in the outcome assessment? | Definitely Low risk of bias | The outcome was assessed using well-established methods and the outcome assessors were adequately blinded to the study group |
| D8: Were all measured outcomes reported? | Probably High risk of bias | There is insufficient information provided about selective outcome reporting |
| Yao et al., (2008) |  |  |
| D1: Was administered dose or exposure level adequately randomized? | Probably High risk of bias | There is insufficient information provided about how subjects were allocated to study groups |
| D2: Was allocation to study groups adequately concealed? | Probably High risk of bias | There is insufficient information provided about allocation to study groups |
| D3: Were experimental conditions identical across study groups? | Probably Low risk of bias | The study included control and treatment group and they used consistent procedures for treatment. |
| D4: Were the research personnel and human subjects blinded to the study group during the study? | Probably High risk of bias | The study does not provide clear information on whether blinding was implemented for research personnel |
| D5: Were outcome data complete without attrition or exclusion from analysis? | Probably High risk of bias | Not reported |
| D6: Can we be confident in the exposure characterization? | Probably Low risk of bias | There is evidence that exposure was consistently administered across treatment groups, however, purity of chemical is not reported. |
| D7: Can we be confident in the outcome assessment? | Probably Low risk of bias | The outcome was assessed using well-established methods |
| D8: Were all measured outcomes reported? | Probably High risk of bias | There is insufficient information provided about selective outcome reporting |
| Schuelert et al., (2010) |  |  |
| D1: Was administered dose or exposure level adequately randomized? | Probably High risk of bias | There is insufficient information provided about how subjects were allocated to study groups |
| D2: Was allocation to study groups adequately concealed? | Probably High risk of bias | There is insufficient information provided about allocation to study groups |
| D3: Were experimental conditions identical across study groups? | Probably Low risk of bias | The study included control and treatment group and they used consistent procedures for treatment. |
| D4: Were the research personnel and human subjects blinded to the study group during the study? | Probably High risk of bias | The study does not provide clear information on whether blinding was implemented for research personnel |
| D5: Were outcome data complete without attrition or exclusion from analysis? | Probably High risk of bias | Not reported |
| D6: Can we be confident in the exposure characterization? | Probably Low risk of bias | There is evidence that exposure was consistently administered across treatment groups, however, purity of chemical is not reported. |
| D7: Can we be confident in the outcome assessment? | Probably Low risk of bias | The outcome was assessed using well-established methods. However, outcome assessor blinding is not mentioned in the study. |
| D8: Were all measured outcomes reported? | Probably High risk of bias | There is insufficient information provided about selective outcome reporting |
| Malek et al., 2022 |  |  |
| D1: Was administered dose or exposure level adequately randomized? | Probably High risk of bias | There is insufficient information provided about how subjects were allocated to study groups |
| D2: Was allocation to study groups adequately concealed? | Probably High risk of bias | There is insufficient information provided about allocation to study groups |
| D3: Were experimental conditions identical across study groups? | Probably Low risk of bias | The study used consistent procedures for treatment and control group. |
| D4: Were the research personnel and human subjects blinded to the study group during the study? | Probably Low risk of bias | The experimenters were blinded to the treatment groups during behavioral assessments using the pressure application measurement test |
| D5: Were outcome data complete without attrition or exclusion from analysis? | Probably High risk of bias | Not reported |
| D6: Can we be confident in the exposure characterization? | Probably Low risk of bias | There is evidence that exposure was consistently administered across treatment groups, however, purity of chemical is not reported. |
| D7: Can we be confident in the outcome assessment? | Probably Low risk of bias | The outcome was assessed using well-established methods. The experimenters were blinded to the treatment groups during behavioral assessments. |
| D8: Were all measured outcomes reported? | Probably High risk of bias | There is insufficient information provided about selective outcome reporting |
| Kong et al., 2016 |  |  |
| D1: Was administered dose or exposure level adequately randomized? | Probably High risk of bias | There is insufficient information provided about how subjects were allocated to study groups |
| D2: Was allocation to study groups adequately concealed? | Probably High risk of bias | There is insufficient information provided about allocation to study groups |
| D3: Were experimental conditions identical across study groups? | Probably Low risk of bias | The experimental conditions were consistent across study groups, with identical culture conditions, and measurement techniques applied uniformly. |
| D4: Were the research personnel and human subjects blinded to the study group during the study? | Probably High risk of bias | The study does not mention whether the researchers administering treatments and measurements were blinded to the experimental groups |
| D5: Were outcome data complete without attrition or exclusion from analysis? | Probably High risk of bias | Not reported |
| D6: Can we be confident in the exposure characterization? | Probably Low risk of bias | Exposure was clearly defined, and the application was consistent across experimental groups |
| D7: Can we be confident in the outcome assessment? | Probably Low risk of bias | The outcome was assessed using well-established methods. The study does not explicitly state whether the outcome assessments were blinded. |
| D8: Were all measured outcomes reported? | Probably High risk of bias | There is insufficient information provided about selective outcome reporting |

# **Table 11:** Risk of bias table for human studies

| Bias | Author judgment | Reason for judgment |
| --- | --- | --- |
| Vela (2021) |  |  |
| R: Bias arising from the randomisation process | Some concern | No information whether allocation sequence concealed |
| D: Bias due to deviations from intended interventions | Low risk | No deviations from intended interventions |
| Mi: Bias due to missing outcome data | Low risk | Small number of lost follow-up patients |
| Me: Bias in measurement of the outcome | Low risk | Appropriate measurement tool |
| S: Bias in selection of the reported result | Some concern | No protocol available |
| O: Overall risk of bias | Some concern |  |
| Campbell (2023) |  |  |
| R: Bias arising from the randomisation process | Some concern | No information whether allocation sequence concealed |
| D: Bias due to deviations from intended interventions | Low risk | No deviations from intended interventions |
| Mi: Bias due to missing outcome data | Some concern | Missing data is not reported |
| Me: Bias in measurement of the outcome | Low risk | Appropriate measurement tool |
| S: Bias in selection of the reported result | Some concern | No protocol available |
| O: Overall risk of bias | Some concern |  |
| Hunter (2018) |  |  |
| R: Bias arising from the randomisation process | Some concern | No detailed information about randomization and allocation sequence concealment is provided |
| D: Bias due to deviations from intended interventions | Low risk | No deviations from intended interventions |
| Mi: Bias due to missing outcome data | High risk | Since this paper is a conference abstract, it does have results from a sensitivity analysis |
| Me: Bias in measurement of the outcome | Low risk | Appropriate method for the outcomes |
| S: Bias in selection of the reported result | Some concern | No protocol available |
| O: Overall risk of bias | High risk | The study was available only as a conference abstract, which further contributed to the lack of sufficient detail |
| Pramhas S (2023) |  |  |
| R: Bias arising from the randomisation process | Low risk | Randomization and allocation sequence concealment is appropriate |
| D: Bias due to deviations from intended interventions | Low risk | No deviations from intended interventions |
| Mi: Bias due to missing outcome data | Low risk | There was no bias from missing data, and a sensitivity analysis was conducted |
| Me: Bias in measurement of the outcome | Low risk | Appropriate method for the outcomes |
| S: Bias in selection of the reported result | Low risk | All the outcomes identified in the protocol are reported |
| O: Overall risk of bias | Low risk |  |
| Heineman (2022) |  |  |
| R: Bias arising from the randomisation process | Low risk | Randomization and allocation sequence concealment is appropriate |
| D: Bias due to deviations from intended interventions | Low risk | No deviations from intended interventions |
| Mi: Bias due to missing outcome data | Some concern | Missing data are not reported |
| Me: Bias in measurement of the outcome | Low risk | Appropriate method for the outcomes |
| S: Bias in selection of the reported result | Some concern | No protocol available |
| O: Overall risk of bias | Some concern |  |
| Bawa (2024) |  |  |
| R: Bias arising from the randomisation process | High risk | No randomization and only one group |
| D: Bias due to deviations from intended interventions | Some concern | Participant aware of their assigned intervention |
| Mi: Bias due to missing outcome data | Low risk | There are no missing data |
| Me: Bias in measurement of the outcome | High risk | Outcome assessor aware of the intervention received by study participant |
| S: Bias in selection of the reported result | Some concern | No protocol available |
| O: Overall risk of bias | High risk |  |

# **Reference**

1. Rockel JS, Maglaviceanu A, Filippini HF, Wasilewski E, Lewis-Bakker MM, Gabrial S, et al. Oral Delivery Of Delta-9-Tetrahydrocannabinol Provides Symptom And Disease Modification In A Mouse Model Of Knee Osteoarthritis. Osteoarthritis and Cartilage. 2023;31:S218.

2. Karuppagounder V, Chung J, Abdeen A, Thompson A, Bouboukas A, Pinamont WJ, et al. Therapeutic Effects of Non-Euphorigenic Cannabis Extracts in Osteoarthritis. Cannabis Cannabinoid Res. 2023;8(6):1030-44.

3. Carmon I, Zecharyahu L, Elayyan J, Meka SRK, Reich E, Kandel L, et al. HU308 Mitigates Osteoarthritis by Stimulating Sox9-Related Networks of Carbohydrate Metabolism. Journal of Bone and Mineral Research. 2020;38(1):154-70.

4. Malek N, Borowczyk J, Kostrzewa M, Pawlowska A, Drukala J, Starowicz K. The Impact of JWH-133 on Articular Cartilage Regeneration in Osteoarthritis Via Metalloproteinase 13-Dependent Mechanism. Cannabis Cannabinoid Res. 2023;8(5):779-89.

5. Verrico CD, Wesson S, Konduri V, Hofferek CJ, Vazquez-Perez J, Blair E, et al. A randomized, double-blind, placebo-controlled study of daily cannabidiol for the treatment of canine osteoarthritis pain. Pain. 2020;161(9):2191-202.

6. Philpott HT, O'Brien M, McDougall JJ. Attenuation of early phase inflammation by cannabidiol prevents pain and nerve damage in rat osteoarthritis. Pain. 2017;158(12):2442-51.

7. Yimam M, O'Neal A, Horm T, Jiao P, Hong M, Rossiter S, et al. Antinociceptive and Anti-Inflammatory Properties of Cannabidiol Alone and in Combination with Standardized Bioflavonoid Composition. Journal of Medicinal Food. 2021;24(9):960-7.

8. Burston JJ, Sagar DR, Shao P, Bai M, King E, Brailsford L, et al. Cannabinoid CB2 receptors regulate central sensitization and pain responses associated with osteoarthritis of the knee joint. PLoS One. 2013;8(11):e80440.

9. Yao BB, Hsieh GC, Frost JM, Fan Y, Garrison TR, Daza AV, et al. <i>In vitro</i> and <i>in vivo</i> characterization of A‐796260: a selective cannabinoid CB<sub>2</sub> receptor agonist exhibiting analgesic activity in rodent pain models. British Journal of Pharmacology. 2008;153(2):390-401.

10. Schuelert N, Zhang C, Mogg AJ, Broad LM, Hepburn DL, Nisenbaum ES, et al. Paradoxical effects of the cannabinoid CB2 receptor agonist GW405833 on rat osteoarthritic knee joint pain. Osteoarthritis and Cartilage. 2010;18(11):1536-43.

11. Gamble L-J, Boesch JM, Frye CW, Schwark WS, Mann S, Wolfe L, et al. Pharmacokinetics, Safety, and Clinical Efficacy of Cannabidiol Treatment in Osteoarthritic Dogs. Frontiers in Veterinary Science. 2018;5.

12. Brioschi FA, Di Cesare F, Gioeni D, Rabbogliatti V, Ferrari F, D’Urso ES, et al. Oral Transmucosal Cannabidiol Oil Formulation as Part of a Multimodal Analgesic Regimen: Effects on Pain Relief and Quality of Life Improvement in Dogs Affected by Spontaneous Osteoarthritis. Animals. 2020;10(9):1505.

13. Gabriele V, Bisanzio D, Riva A, Meineri G, Adami R, Martello E. Long-term effects of a diet supplement containing <i>Cannabis sativa</i> oil and <i>Boswellia serrata</i> in dogs with osteoarthritis following physiotherapy treatments: a randomised, placebo-controlled and double-blind clinical trial. Natural Product Research. 2023;37(11):1782-6.

14. Mejia S, Duerr FM, Griffenhagen G, McGrath S. Evaluation of the Effect of Cannabidiol on Naturally Occurring Osteoarthritis-Associated Pain: A Pilot Study in Dogs. J Am Anim Hosp Assoc. 2021;57(2):81-90.

15. Vela J, Dreyer L, Petersen KK, Arendt-Nielsen L, Duch KS, Kristensen S. Cannabidiol treatment in hand osteoarthritis and psoriatic arthritis: a randomized, double-blind, placebo-controlled trial. Pain. 2022;163(6):1206-14.

16. Pramhas S, Thalhammer T, Terner S, Pickelsberger D, Gleiss A, Sator S, et al. Oral cannabidiol (CBD) as add-on to paracetamol for painful chronic osteoarthritis of the knee: a randomized, double-blind, placebo-controlled clinical trial. The Lancet Regional Health - Europe. 2023;35:100777.

17. Heineman JT, Forster GL, Stephens KL, Cottler PS, Timko MP, DeGeorge BR, Jr. A Randomized Controlled Trial of Topical Cannabidiol for the Treatment of Thumb Basal Joint Arthritis. J Hand Surg Am. 2022;47(7):611-20.

18. Hunter D, Oldfield G, Tich N, Messenheimer J, Sebree T. Synthetic transdermal cannabidiol for the treatment of knee pain due to osteoarthritis. Osteoarthritis and Cartilage. 2018;26:S26.

19. Campbell CM, Mun CJ, Hamilton KR, Bergeria CL, Huhn AS, Speed TJ, et al. Within-subject, double-blind, randomized, placebo-controlled evaluation of combining the cannabinoid dronabinol and the opioid hydromorphone in adults with chronic pain. Neuropsychopharmacology. 2023;48(11):1630-8.

20. Bawa Z, Lewis D, Gavin PD, Libinaki R, Joubran L, El-Tamimy M, et al. An open-label feasibility trial of transdermal cannabidiol for hand osteoarthritis. Scientific Reports. 2024;14(1):11792.
